# Rhodium-Catalyzed Synthesis of Organosulfur Compounds Involving S-S Bond Cleavage of Disulfides and Sulfur

**DOI:** 10.3390/molecules25163595

**Published:** 2020-08-07

**Authors:** Mieko Arisawa, Masahiko Yamaguchi

**Affiliations:** Department of Organic Chemistry, Graduate School of Pharmaceutical Sciences, Tohoku University, Aoba, Sendai 980-8578, Japan; yama@m.tohoku.ac.jp

**Keywords:** rhodium, catalysis, synthesis, organosulfur compounds, S-S bond cleavage, chemical equilibrium, reversible reaction

## Abstract

Organosulfur compounds are widely used for the manufacture of drugs and materials, and their synthesis in general conventionally employs nucleophilic substitution reactions of thiolate anions formed from thiols and bases. To synthesize advanced functional organosulfur compounds, development of novel synthetic methods is an important task. We have been studying the synthesis of organosulfur compounds by transition-metal catalysis using disulfides and sulfur, which are easier to handle and less odiferous than thiols. In this article, we describe our development that rhodium complexes efficiently catalyze the cleavage of S-S bonds and transfer organothio groups to organic compounds, which provide diverse organosulfur compounds. The synthesis does not require use of bases or organometallic reagents; furthermore, it is reversible, involving chemical equilibria and interconversion reactions.

## 1. Introduction

### 1.1. Structure and Reactivity of Organic Disulfides

Organosulfur compounds containing C-S bonds are widely used for the manufacture of drugs and materials. Compared with organic compounds containing oxygen, which is another group 16(6A) element, different properties appear owing to the large size and polarizability of sulfur atoms. A characteristic feature of inorganic and organic sulfur compounds is the involvement of different oxidation states (between −2 and +6) of sulfur atoms, which give rise to diverse sulfur functional groups [1]. Thiols (RSH) and sulfonic acids (RSO_3_H) are organosulfur compounds with low and high oxidation states, respectively. Sulfenic acids (RSOH) and sulfinic acids (RSO_2_H) exhibit intermediate oxidation states. These sulfur acids can form ester and amide derivatives. Elemental sulfur in the oxidation state of 0 is a convenient source of organosulfur compounds.

Among organic functional groups containing sulfur, disulfides (RS-SR) with S-S bonds are of interest. In contrast to peroxides (RO-OR) with O-O bonds, disulfides are stable and exhibit significantly different reactivities. The bond energy of S-S bonds is 226 kJ mol^−1^ (for S_8_) [2,3,4,5], which is the highest among the X-X bonds of the group 16 elements: O-O, 142 kJ mol^−1^; Se-Se, 172 kJ mol^−1^; and Te-Te, 150 kJ mol^−1^. Disulfides have a molecular structure with a dihedral angle of C-S-S-C of approximately 90° in the most stable conformation.

Proteins and peptides contain disulfides that form their three-dimensional structures [6,7,8,9] Disulfides in proteins are found predominantly in secreted extracellular proteins, and thiols are preserved in the cytosol in a reductive environment. It is known that the functions of proteins can be switched via the cleavage or formation of disulfides is known [10,11]. Disulfides are found in some small molecules that are biologically active natural products [12].

Sulfides, disulfides, and polysulfides are important functional groups in synthetic rubber [13]. Natural rubber is treated with sulfur to convert it into materials with a large range of hardness, elasticity, and mechanical durability, in which sulfur atoms form cross-linking bridges between polymer chains in a process called vulcanization.

**Figure 1:**
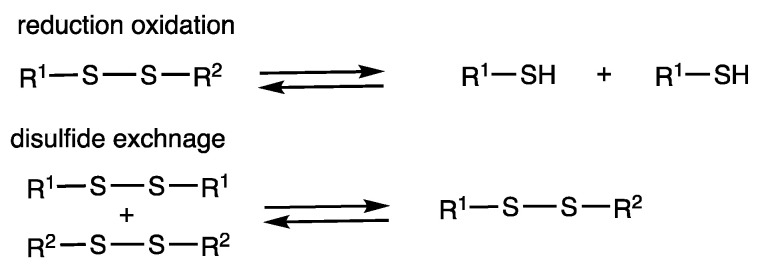
Interconversion reactions of disulfides/thiols by reduction/oxidation and chemical equilibrium under disulfide exchange.

**Scheme 1:**
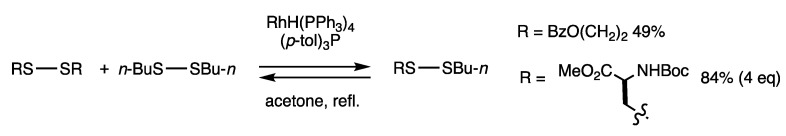
Disulfide exchange reaction.

An important reversible reaction of disulfides is their reduction to thiols, which may be oxidized to disulfides (Figure 1). Such reactivity has been utilized in biological systems and also in synthetic systems for molecular switching [14]. Various reactions have been reported for the interconversion. The exchange reaction of S-S bonds between disulfides is another important reversible reaction (Scheme 1) [15,16]. The reaction of two different disulfides produces a statistical 1:1:2 mixture of two symmetric disulfides and an unsymmetric disulfide under chemical equilibrium when their thermodynamic stabilities are comparable.

Both reduction/oxidation and exchange reactions of disulfides can be used for a molecular switching function. A characteristic aspect of disulfide exchange reactions compared with reduction/oxidation reactions is that direct one-step interconversion proceeds without forming thiols. This makes procedures simple, and various transformations that are incompatible in the presence of thiols can be conducted. Basic conditions are often employed for disulfide exchange reactions, which involve the nucleophilic attack of thiolate anions on S-S bonds via an S_N_2 mechanism. Photoirradiation is effective for the exchange reaction of aromatic disulfides, which involves the homolytic cleavage of S-S bonds generating thiyl radicals. Acidic conditions can also be employed. The reactivity of disulfides depends on their substituents. Aromatic disulfides are easier to dissociate than aliphatic disulfides, which reflects the relative dissociation energies of PhS-SPh (230 kJ mol^−1^) and MeS-SMe (309 kJ mol^−1^) [3].

### 1.2. Synthesis of Organosulfur Compounds Using Disulfides

Synthesis of organosulfur compounds has generally been conducted using thiols, and a typical method is a substitution reaction with organohalogen compounds in the presence of a base [17,18,19,20]. The roles of bases are to form highly reactive thiolate anions and to neutralize hydrogen halides formed as byproducts. The neutralization reaction is significantly exothermic and promotes the reaction according to the Bell–Evans–Polanyi principle [17].

The use of disulfides in the substitution reaction of organohalogen compounds has been rare. This is because disulfides are neutral compounds and are less reactive than thiolate anions. Consider a hypothetical substitution reaction of an organohalogen compound and a disulfide to provide a sulfide containing a C-S bond. Formally, the reaction is accompanied by the formation of a sulfenyl halide, which is thermodynamically unstable and makes the reaction thermodynamically unfavorable. The use of disulfides in organic synthesis, however, can have several advantages over the use of thiolate anions: (1) disulfides are stable and easy to handle; (2) they are less odiferous; (3) they can be activated by various methods, including the use of acids, bases, radicals, metals, and photoirradiation; and (4) they do not form metal halide byproducts. In addition, characteristic reactivities of disulfides can appear, which thiols do not have. As such, special methods are needed to utilize disulfides in the synthesis of organosulfur compounds. An example was reported in the oxidation–reduction condensation of bi(2-pyridyl) disulfide and a carboxylic acid in the presence of triphenylphosphine to provide a 2-pyridylthio ester [21]. The reaction is thermodynamically favorable because of the exothermic oxidation reaction of triphenylphosphine to the corresponding oxide. Thus, it is reasonable to consider that disulfides can be used as substrates in organic synthesis and that the reactions can proceed in the absence of bases.

### 1.3. Rhodium-Catalyzed Synthesis of Organosulfur Compounds Using Disulfides

Transition-metal catalysis for the synthesis of organosulfur compounds has attracted interest; however, this interest has been limited. In particular, the use of disulfides has been rare, with the only exceptions of addition reactions to alkenes and alkynes originally reported by Ogawa [22], Beletskaya [23], and Mitsudo [24]. The lack of such synthetic methods for organosulfur compounds is due to the strong bonding between transition metals and sulfur atoms, which does not readily allow the liberation of the metals and products; as a result, the catalyst cannot be regenerated. Methods are required to overcome the relatively unreactive nature of neutral disulfides and to prevent catalyst deactivation by (1) the development of highly active catalysts and (2) the judicious choice of substrates and products that produce exothermic reactions. 

**Figure 2:**
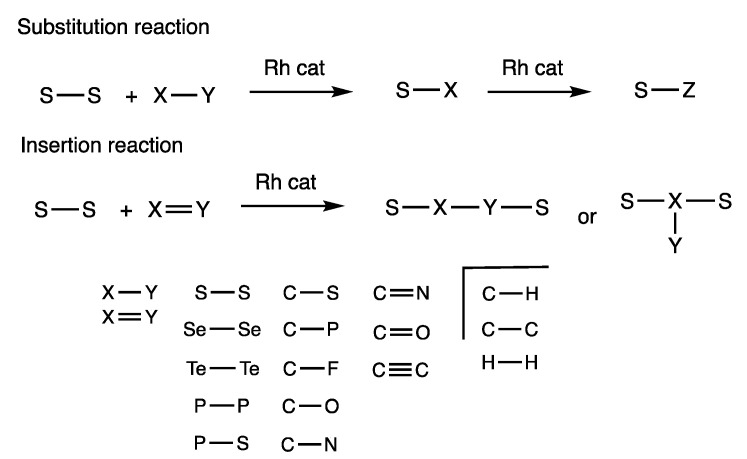
Diverse reactivity of substitution and insertion reactions of the S-S bond in disulfides with various other chemical bonds.

We have found that rhodium complexes catalyze various substitution and insertion reactions using disulfides, which indicates that sulfur ligands on rhodium atoms liberate organosulfur compounds with regeneration of the rhodium catalyst. In this article, a substitution reaction is defined as the transformation of a S-S bond and an X-Y single bond to form a S-X bond; an insertion reaction is defined as the transformation of a S-S bond and an X=Y multiple bond to form a S-X-S subunit with one atom of X inserted or a S-X-Y-S subunit with two atoms of X-Y inserted (Figure 2). We describe in this article that rhodium-catalyzed activation of S-S bonds can be applied to a broad range of chemical transformations with different organic compounds containing S-S, Se-Se, Te-Te, P-P, and P-S heteroatom bonds, along with C-S, C-P, C-F, C-N, C-O, C-H, C-C, and H-H bonds. Unsaturated C=O, C≡C, and C=N bonds can also participate in the rhodium-catalyzed reactions of S-S bonds.

A characteristic feature of rhodium catalysis is its capability to activate C-H bonds [25], which is utilized here for C-S bond formation of 1-alkynes, nitroalkanes, malonates, α-phenylketones, ketones, aldehydes, and heteroaromatic compounds. Activation of C-C bonds in ketones and H-H bonds in hydrogen is also shown.

**Scheme 2:**
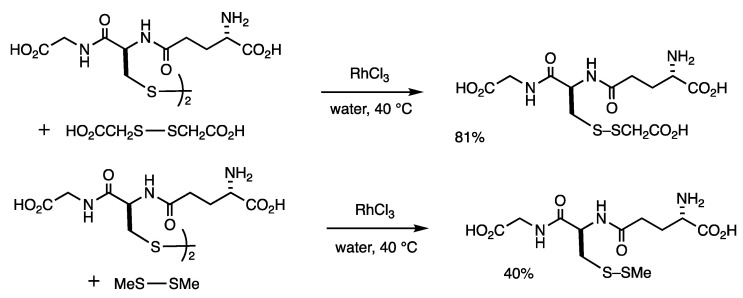
Disulfide exchange reaction of glutathione in water.

**Scheme 3:**
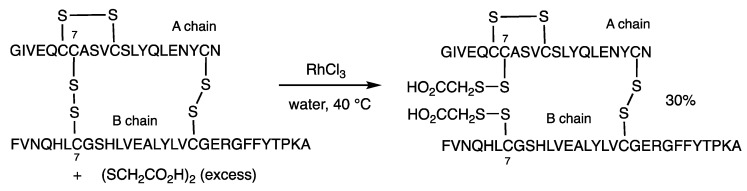
Disulfide exchange reaction of insulin.

Another notable property of rhodium-catalyzed synthesis using disulfides is its applicability to reactions in water. Peptides and proteins containing the cysteine moiety can be modified in water without protecting groups (Schemes 2, 3, and 16). These results indicate a high tolerance of functional groups in rhodium catalysis, which selectively activates disulfides in the presence of a large number of oxygen- and nitrogen-containing functional groups.

The most stable form of elemental sulfur is the eight-membered S_8_ ring containing S-S bonds, which is produced by desulfurization of petroleum. Sulfur exhibits chemical reactivities similar to those of disulfides, but different chemical reactivities also appear. This is because disulfides contain sulfur atoms bonded to one sulfur atom and one organic group; sulfur contains sulfur atoms bonded to two sulfur atoms. Although organic synthesis using sulfur has attracted attention [26], it has generally been conducted by thermal reactions involving sulfur radicals and by nucleophilic reactions using highly reactive main group metal reagents. In this study, synthesis of organosulfur compounds using disulfides under rhodium catalysis is extended to syntheses using sulfur.

Along with the development of rhodium-catalyzed synthesis of organosulfur compounds involving S-S bond cleavage, we have studied the synthesis of organophosphorus compounds involving P-P bond cleavage. This comparative study of organoheteroatom compounds with elements adjacent on the periodic table is a novel approach. 

### 1.4. Reversible Nature of Rhodium-Catalyzed Reactions of Disulfides

**Figure 3:**
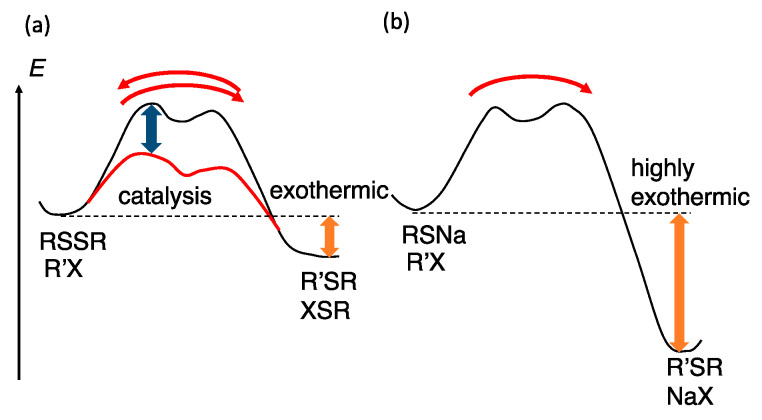
(**a**) Chemical equilibrium of rhodium-catalyzed reactions using disulfides and a cosubstrate; (**b**) highly exothermic irreversible reaction using metal thiolates.

Transition-metal-catalyzed reactions using disulfides to provide organosulfur compounds often reach chemical equilibria and are reversible. This behavior implies that the relative thermodynamic stabilities of substrates and products are similar and the energy barrier is low (Figure 3a). Consequently, shifting the chemical equilibrium toward the desired product is critical to obtaining high yields. In this study, we developed several methods for that purpose: (1) structures of substrates and products are selected to provide exothermic reactions; (2) chemical equilibrium is shifted using a larger amount of one substrate; (3) chemical equilibrium is shifted, removing a volatile product; (4) appropriate combinations of cosubstrates and coproducts are developed to provide exothermic reactions; (5) a product is converted to thermodynamically stable form; and (6) the desired product is removed from an equilibrium mixture by silica nanoparticle precipitation. In this way, the nature of the synthetic reactions for organosulfur compounds, presented herein, is significantly different from that of conventional irreversible reactions using thiolate anions, which involve strong exothermic reactions using bases (Figure 3b).

It is thought that chemical equilibrium is not favorable in organic synthesis because chemical yields are governed by the relative thermodynamic stability of substrates and products. We show in this article that the use of chemical equilibrium has intrinsic synthetic advantages: (1) chemical equilibrium is energy-saving because it does not require a strong exothermic reaction and it involves a small energy barrier; (2) chemical equilibrium can provide different products by shifting the equilibrium through the control of reaction conditions; (3) regeneration of substrates from products is easy; (4) catalysis is effective for both forward and backward reactions, which can be used to promote the reaction; (5) chemical equilibria generally do not form inorganic byproducts, which is inevitable in exothermic reactions using bases. It should also be noted that the recovery of bases from inorganic byproducts is tedious and energy-consuming. 

A novel concept is derived from the reversible nature of the rhodium-catalyzed synthesis of organosulfur compounds under chemical equilibrium. Catalysts can cleave C-S bonds of products, which implies that, in the presence of suitable acceptors, products can be converted to other organosulfur compounds: rhodium complexes can activate S-X bonds in the products and convert then into compounds with S-Z bonds (Figure 2). Such examples are described for the reactions of 1-thioalkynes and thioesters in Section 7. Our working hypothesis on the mechanisms of rhodium-catalyzed reactions of disulfides is the involvement of oxidative addition of low-valent rhodium to form S-Rh-S species, which is followed by substitution or insertion by other organic groups. Chemical equilibrium indicates that all processes are reversible.

Organosulfur compounds can be used as alkylating reagents analogous to organohalogen compounds. This concept is inferred from the bond energy of C-S (272 kJ mol^−1^), which is comparable to that of C-Br (285 kJ mol^−1^) [1]. Organothio groups can be used as leaving groups in substitution reactions, and they can exhibit different properties from halogen groups. Sulfur leaving groups have divalent sulfur atoms, whereas halogen leaving groups have monovalent atoms, and the properties of the sulfur leaving groups can be tuned by the organic groups. In addition, substitution reactions using sulfur leaving groups are reversible under rhodium catalysis; such examples are described in Section 7. It should be noted that the bond energy of C-S is comparable to that of C-P (264 kJ mol−1) and that organosulfur and organophosphorus compounds are interconvertible (Schemes 14, 18, and 33).

**Figure 4:**
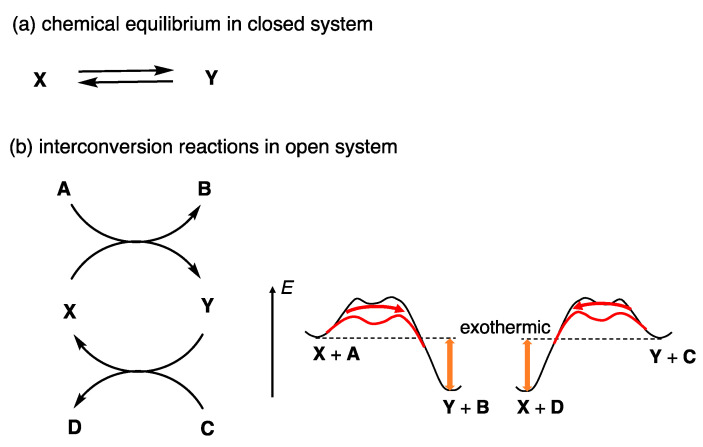
(**a**) Chemical equilibrium in a closed system between **X** and **Y**. (**b**) Interconversion reaction in an open system between **X** and **Y**, in which the relative thermodynamic stability of substrates and products is inverted and induces **X** + **A** → **Y** + **B** and **Y** + **C** → **X** + **D** reactions.

The above describes the reversibility of reactions under chemical equilibrium between **X** and **Y** in a closed system, in which no matter is exchanged with the surroundings but energy is exchanged (Figure 4a). Such reactions are indicated in this article by two straight arrows pointing in opposite directions. A reversible reaction in an open system can also be considered, in which both matter and energy are exchanged. Substrate **X** and product **Y** are interconverted by the addition of reagents **A** and **C**, which provide the **X** + **A** → **Y** + **B** and **Y** + **C** → **X** + **D** reactions, respectively (Figure 4b). Addition of **A** and **C** makes these reactions exothermic, and catalysis reduces the energy barrier, which accelerates these reactions. Such reactions are called interconversion reactions in this article and are expressed by curved arrows pointing in opposite directions. Many of the reactions described in this article are reversible and involve catalysis either under chemical equilibria or interconversion reactions. Reversibility in synthetic reactions provides a novel concept in chemistry that has some similarity with biological systems.

The model of interconversion reactions in an open system between **X** and **Y** provides another interesting aspect in the development of synthetic reactions (Figure 4b). When the model is analyzed in terms of input and output, the **A** + **C** → **B** + **D** reaction can be considered. Such reactions can be developed, as shown in this article (Schemes 5, 7, and 14). **X** and **Y** can be used as catalysts for the **A** + **X** → **B** + **Y** and **Y** + **C** → **X** + **D** reactions proceeding under the same conditions. 

One of our purposes in the study of transition-metal-catalyzed synthesis of organosulfur compounds is the development of biologically active compounds. Heteroatoms are essential for biological functions, as shown by the huge amounts of nitrogen and oxygen atoms used by living things. In contrast, the use of sulfur is limited mostly to amino acids, and the development of biologically active organosulfur compounds, which exhibit exotic properties for living things, is an interesting subject. The transition-metal-catalyzed synthetic method discussed herein has indeed provided biologically active organosulfur compounds [26,27].

Our previous review articles on the transition-metal-catalyzed synthesis of organosulfur compounds focused on the development of exothermic reactions [28,29], the synthesis and properties of bis(heteroaryl) compounds [26,27], the use of elemental sulfur [30], and the P-P bond cleavage reactions [31]. The present article describes an overview of our studies that began in the early 2000s and involve classification of the chemical reactions with S-S bond cleavage and organothio transfer. In addition, emphasized herein are the chemical equilibria and interconversion reactions provided by the reversible nature of the rhodium-catalyzed synthesis.

## 2. Rhodium-Catalyzed Exchange Reactions of Disulfides

Disulfides can exchange organothio groups under acidic or basic conditions and when exposed to heat or photoirradiation. We found that disulfide exchange reactions proceed efficiently in the presence of a rhodium complex [32]. A mixture of RhH(PPh_3_)_4_, sulfonic acid, and phosphine promotes catalysis of the disulfide exchange reaction, which proceeds rapidly under acetone reflux (Scheme 1). Dibutyl disulfide and bis(2-benzoyloxyethyl) disulfide were reacted in refluxing acetone in the presence of RhH(PPh_3_)_4_ (3 mol%), trifluoromethanesulfonic acid (6 mol%), and (*p*-tol)_3_P (12 mol%), and 2-benzoyloxyethyl butyl disulfide was obtained in 49% yield. The reaction is applicable to both aliphatic and aromatic disulfides, and it proceeds at a much lower temperature than in the conventional heating method. A chemical equilibrium was rapidly reached within 15 min, which included two symmetric disulfides and one asymmetric disulfide at a statistical 1:1:2 ratio. Chemical equilibrium was confirmed by the reverse reaction of an unsymmetric disulfide. The method is applicable to the exchange of disulfides, diselenides/ditellurides, and other heteroatom compounds of group 16 elements. The proposed mechanism involves the initial oxidative addition of a low-valent rhodium complex and a disulfide. The subsequent oxidative addition of another disulfide or ligand exchange of the organothio group occurs, and the disulfide is liberated by reductive elimination with the regeneration of the catalyst.

**Figure 5:**
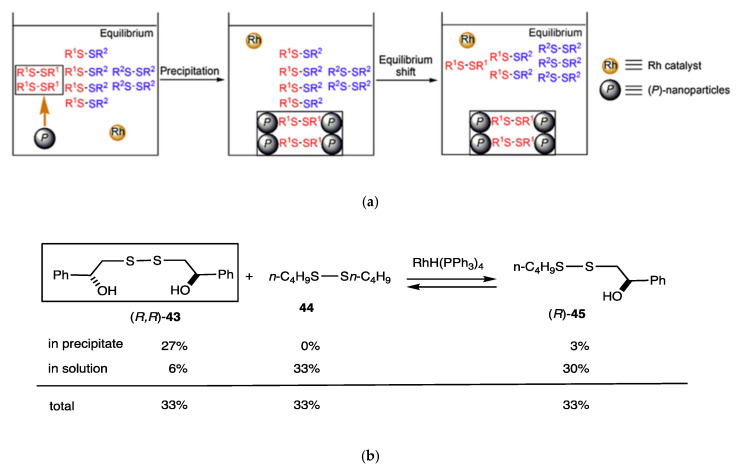
(**a**) Equilibrium shift in disulfide exchange reaction induced by the precipitation of silica (*P*)-nanoparticles. (**b**) Equilibrium shift in the reaction of (*R*, *R*)-**43**, **44**, and (*R*)-**45**.

When one product is removed from the solution of the disulfide exchange reaction, the chemical equilibrium can be shifted (Figure 5a) [33]. We developed a silica nanoparticle precipitation method, in which nanoparticles precipitated from solution with concomitant molecular recognition and adsorption of molecules. Silica (*P*)-nanoparticles of 70 nm mean diameter grafted with (*P*)-helicene were employed. When butyl (*R*)-(hydroxyphenylmethyl) disulfide (*R*)-**45** was treated with RhH(PPh_3_)_4_ (20 mol%), trifluoromethanesulfonic acid (40 mol%), and (*p*-tol)_3_P (80 mol%) in chlorobenzene for 24 h in the presence of silica (*P*)-nanoparticles, precipitates containing (*R*,*R*)-bis(hydroxyphenylmethyl) disulfide (*R*, *R*)-**43** (27%) and (*R*)-**45** (3%) were formed (Figure 5b). The high preference for (*R*, *R*)-**43** is notable, and no dibutyl disulfide **44** was contained in the precipitates. The solution phase contained (*R*, *R*)-**43** (6%), (*R*)-**45** (30%), and **44** (33%). The composition of disulfides in the precipitates deviates considerably from that of the initial chemical equilibrium with (*R*, *R*)-**43**:(*R*)-**45**:**44** = 1:2:1. It should also be noted that most of the butylthio group remained in solution.

The disulfide exchange reaction of hydrophilic disulfides occurs in water when using the RhCl_3_ catalyst (Scheme 2) [34]. When glutathione disulfide and glycolic acid disulfide (4 equivalents) were treated with RhCl_3_ (10 mol%) in water at 40 °C for 1 h, methylthiolated glutathione was obtained in 81% yield. The reverse reaction confirmed the involvement of chemical equilibrium. In addition, the composition under chemical equilibrium could be changed by the addition of a disulfide, which indicated that catalysis occurred after reaching chemical equilibrium. This method can be applied to the reaction of dimethyl disulfide, which is not water-soluble. A two-phase system of glutathione disulfide in water and excess dimethyl disulfide was treated with RhCl_3_ (10 mol%) at 40 °C for 1 h, and methylthiolated glutathione was obtained in 40% yield.

The RhCl_3_-catalyzed method was applied to insulin containing three S-S bonds, and the reaction with excess thioglycolic acid preferentially exchanged the disulfide at the 7 positions in both A and B chains (Scheme 3) [35]. The product was obtained in 30% yield at room temperature for 1 h using RhCl_3_ (300 mol%) with recovery of insulin in 70% yield. The rhodium-catalyzed method in water tolerates various functional groups, including amides, amines, carboxylic acids, alcohols, and phenols, because of the higher affinity of rhodium for sulfur atoms in the presence of nitrogen and oxygen functional groups.

## 3. Rhodium-Catalyzed Substitution Reactions Using Disulfides

The S-S bonds of disulfides are reversibly cleaved under rhodium catalysis, as indicated by the disulfide exchange reaction. Rhodium complexes also cleave C-S and C-H bonds in organic compounds, and combinations of these bond cleavage reactions provide various chemical transformations for the synthesis of organosulfur compounds, including thioesters, α-thioketones, 1-thioalkynes, aryl sulfides, and dithiophosphinates.

### 3.1. Substitution Reactions of Thioesters

**Scheme 4:**
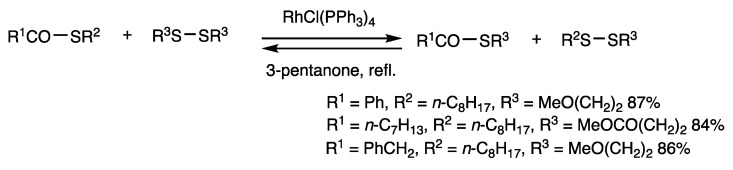
Organothio exchange reaction of thioesters.

Thioesters are excellent substrates for rhodium-catalyzed reactions and provide various acyl derivatives by their reactions with different substrates. Organothio exchange reaction of thioesters occurs with disulfides (Scheme 4) [36]. *S*-Octyl benzothioate and bis(2-ethoxyethyl) disulfide (4 equivalents) were reacted in the presence of RhCl(PPh_3_)_3_ (2.5 mol%) at 3-pentanone reflux for 1.5 h, and *S*-(2-ethoxyethyl) benzothioate was obtained in 87% yield. Chemical equilibrium was determined by the reverse reaction and formation of a statistical 1:2:1 mixture employing 1 equivalent of disulfides. It was also observed that the reaction of *S*-methyl thioesters and disulfides (4 equivalents) gave higher yields of the exchange products under 1,2-dichlorobenzene reflux because of the removal of volatile dimethyl disulfide from the reaction mixture. These results indicate facile cleavage of C-S bonds in thioesters by rhodium catalysis.

**Scheme 5:**
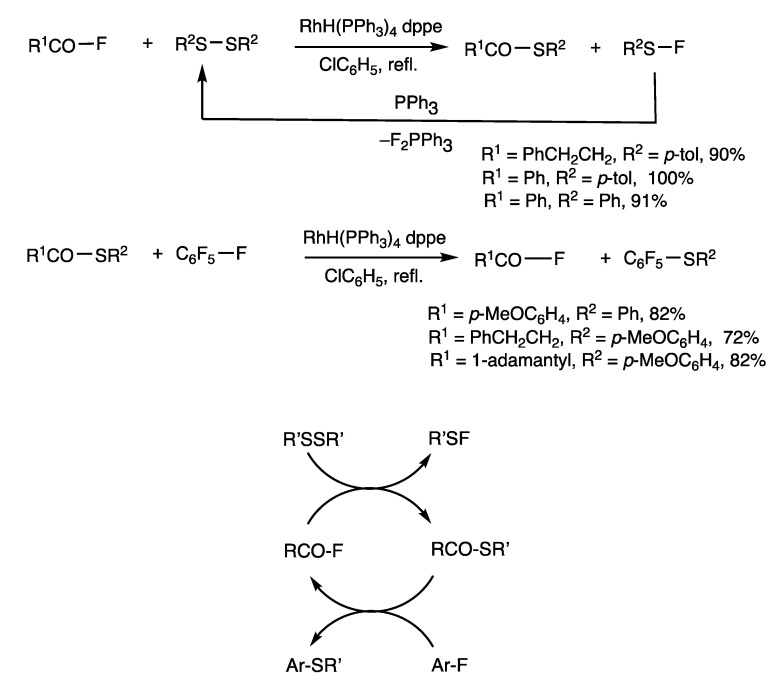
Interconversion between acid fluorides and thioesters.

Acid fluorides show high reactivity under rhodium-catalyzed conditions (Scheme 5) [37]. When benzoyl fluoride, bis(*p*-tolyl) disulfide, and triphenylphosphine were reacted in the presence of RhH(PPh_3_)_4_ (1 mol%) and 1,2-diphenylphosphinoethane (dppe) (2 mol%) in refluxing THF for 2 h, *S*-(*p*-tolyl) benzothioate was obtained in 100% yield. Formally, the reaction provides unstable sulfenyl fluoride with disulfide regeneration. The role of triphenylphosphine is to trap fluorides to form phosphine difluoride with disulfide regeneration, which results in an exothermic reaction suitable for catalysis.

The rhodium-catalyzed method was also employed in the synthesis of acid fluorides from thioesters (Scheme 5) [37]. When *S*-(*p*-tolyl) benzothioate and hexafluorobenzene were reacted in the presence of RhH(PPh_3_)_4_ (2.5 mol%) and dppe (5 mol%) in refluxing chlorobenzene, benzoyl fluoride was obtained in 94% yield in addition to 1,4-di(*p*-tolylthio)-2,3,5,6-tetrafluorobenzene. This is a noteworthy fluorinating reaction using stable neutral aromatic fluorides. Chemical equilibrium also occurs between an acid fluoride and a thioester in a closed system under rhodium catalysis.

The above results indicate involvement of interconversion reactions in an open system between thioester and acid fluoride catalyzed by rhodium: Acid fluorides are converted to thioesters by adding disulfides, and thioesters are converted to acid fluorides by adding hexafluorobenzene. The addition of external reagents inverts the relative thermodynamic stability and promotes the interconversion reactions (Figure 4). It was considered that a R’SSR’ + ArF → Ar-SR’ + R’S-F reaction may also occur; this reaction is described later (Scheme 12).

**Scheme 6:**
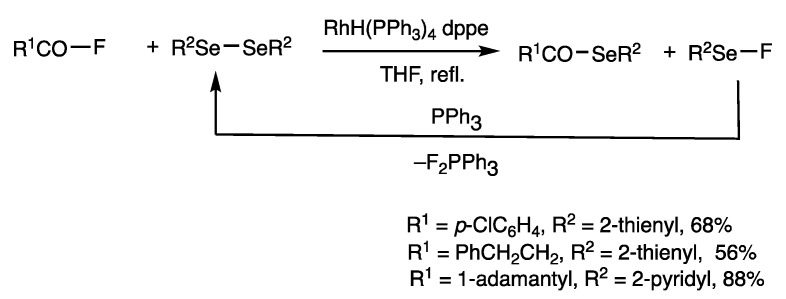
Synthesis of selenoesters.

Rhodium-catalyzed reactions of disulfides can be applied to diselenides (Scheme 6) [38]. Bis(2-pyridyl) diselenide and 1-adamantanecarbonyl fluoride were reacted in the presence of RhH(PPh_3_)_4_ (2.5 mol%) and dppe (5 mol%) in refluxing chlorobenzene, and 1-adamantanecarbonyl 2-pyridylselenoester was obtained in 88% yield. This method was used for the synthesis of heteroaryl selenoesters, which are generally less stable than aryl thioesters. The heteroaryl compounds can be used for the synthesis of novel organoselenium compounds.

### 3.2. Substitution Reactions of 1-Alkynes

Rhodium complexes can activate C-H bonds of organic molecules, and this reaction is employed here for thiolation reactions using disulfides [30]. The reaction of a S-S bond in a disulfide and a C-H bond in an organic compound formally provides an organosulfur compound with a C-S bond and a thiol with a S-H bond. The thiol can be oxidized to a disulfide in the presence of oxygen, a reaction that is also catalyzed by rhodium complexes. 

**Scheme 7:**
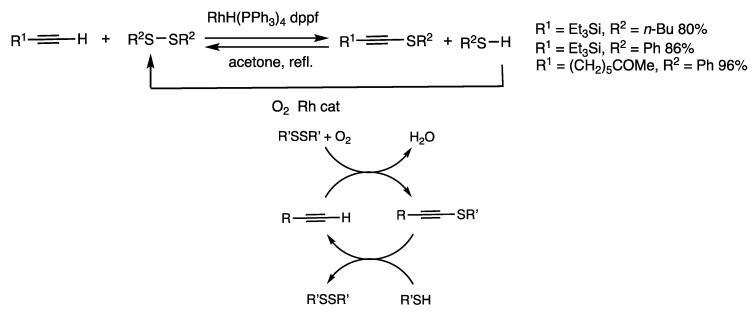
Organothiolation reaction of 1-alkynes.

When 1-(triethylsilyl)-acetylene was treated with dibutyl disulfide in the presence of RhH(PPh_3_)_4_ (2 mol%) and diphenylphosphinoferrocene (dppf) (3 mol%) for 1 h in refluxing acetone, 1-butylthio-2-triethylsilylacetylene was obtained in 80% yield, which was accompanied by a thiol (Scheme 7) [39]. The reaction of a thiol and a 1-thioacetylene formed the original 1-alkyne under argon atmosphere. 1-Alkynes/disulfides and 1-thioalkyne/thiols are under chemical equilibrium in the presence of rhodium catalysis. The rhodium complex also rapidly oxidizes thiols to disulfides and water in the presence of a trace amount of oxygen (Scheme 7 and Scheme 23). In combination with the oxidation reaction under air, the thiolation reaction of 1-alkynes proceeds in an energetically downhill manner to provide 1-thioalkynes in higher yields. The interconversion reactions proceed between 1-alkynes and 1-thioalkynes.

**Scheme 8:**
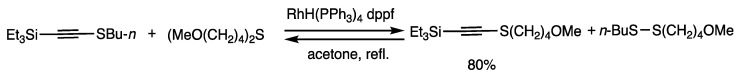
Organothio exchange reaction of 1-thioalkyne.

A rhodium complex catalyzes the organothio exchange reaction of 1-thioalkynes with disulfides, the observation of which confirmed the C-S bond cleavage by rhodium catalysis (Scheme 8) [39]. In principle, diverse 1-thialkynes can be obtained by this method using different disulfides. 

### 3.3. Substitution Reactions of Active Methylene Compounds

**Scheme 9:**
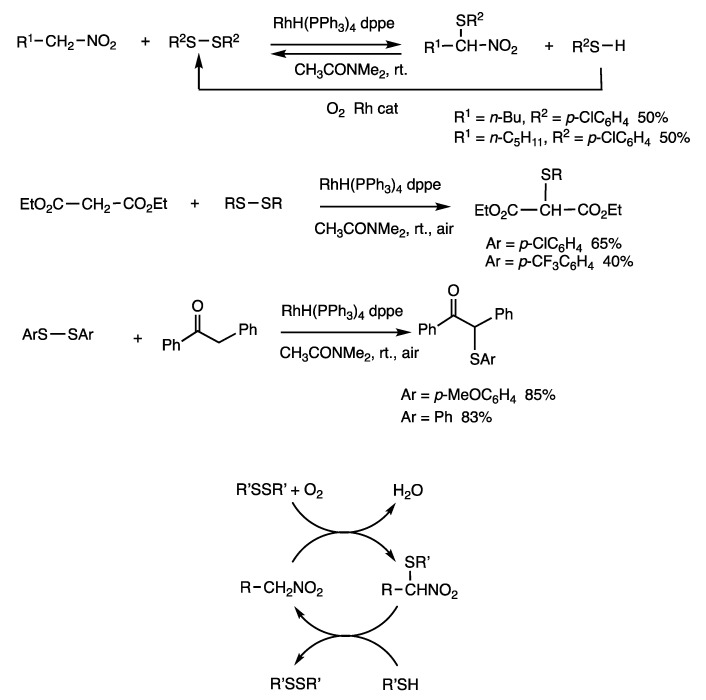
Organothiolation reactions of active methylene compounds.

Active methylene compounds, which include nitroalkanes, malonate, and benzyl ketones, with acidic hydrogen atoms are thiolated with disulfides under rhodium catalysis (Scheme 9) [40]. When 1-nitropentane was treated with bis(*p*-chlorophenyl) disulfide in *N*, *N*-dimethylacetamide (DMA) in air at room temperature for 3 h in the presence of RhH(PPh_3_)_4_ (5 mol%) and dppe (10 mol%), 2-(*p*-chlorophenyl) nitropentane was produced in 50% yield. The reaction without air is under chemical equilibrium, and α-thiolated nitroalkanes are thermodynamically unfavorable. Accordingly, treatment of an α-thiolated nitroalkane with a thiol quantitively provided the original nitroalkane. Air/oxygen converts thiols to disulfides and water in an exothermic reaction. Diethyl malonate and 1,2-diphenyl-1-ethanone were also reacted with aromatic disulfides in air to provide organothiolated compounds. 

### 3.4. Substitution Reactions of Ketones and Heteroarenes

α-Thioketones efficiently undergo C-S substitution reactions in the presence of rhodium catalysts. In combination with the C-H cleavage reaction catalyzed by rhodium complexes, organothiolation of various organic compounds, including ketones and heteroarenes, occurs.

**Scheme 10:**
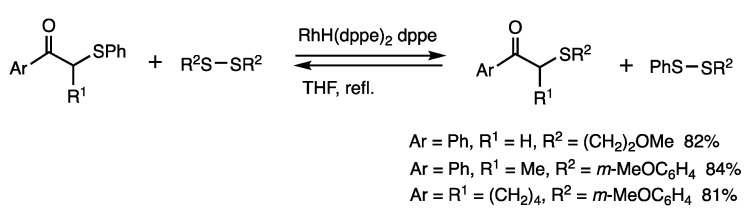
Organothio exchange reaction of α-thioketones.

It was determined that organothio exchange reactions of α-thioketones with disulfides proceeded with the involvement of C-S bond cleavage by rhodium catalysis (Scheme 10) [41]. When an α-phenylthioacetophenone was treated with bis(3-methoxypropyl) disulfide (3 equivalents) in the presence of RhH(PPh_3_)_4_ (1 mol%) and dppe (2 mol%) in refluxing THF for 1–2 h, α-(3-methoxypropyl) acetophenone was obtained in 82% yield. The reverse reaction under the same conditions indicated the involvement of chemical equilibrium. The exchange reactions of organothio groups using different disulfides under rhodium catalysis provide diverse derivatives starting from a single α-thioketone.

**Scheme 11:**
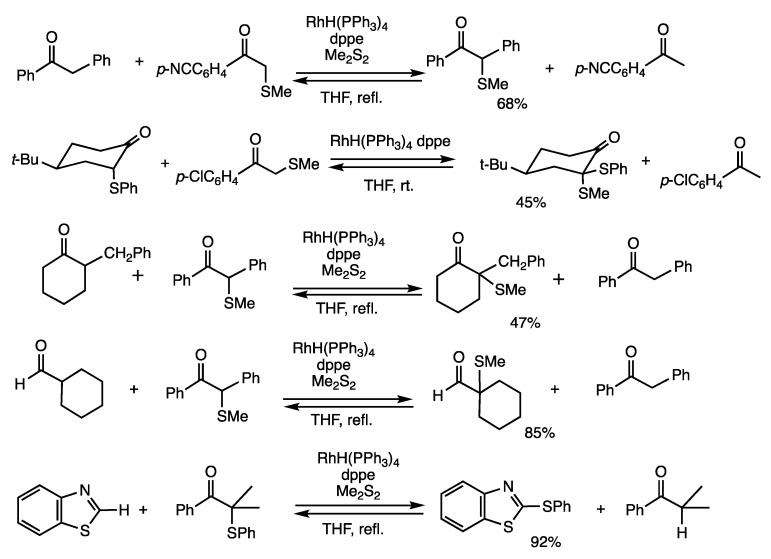
Organothiolation reactions of ketones and benzothiazole.

α-Thioketones can be used as organothiolating reagents of organic compounds via organorhodium intermediates with C-Rh-S subunits (Scheme 11). A methylthio transfer reaction occurs between different ketones at the α-position, in which a catalytic amount of dimethyl disulfide significantly promotes the reaction [42]. When α-methylthio-*p*-cyanoacetophenone and 1,2-diphenylethanone were reacted in refluxing THF for 3 h in the presence of RhH(PPh_3_)_4_ (4 mol%), dppe (8 mol%), and dimethyl disulfide (12 mol%), 2-methylthio-1,2-diphenylethanone was obtained in 68% yield. The methylthio group moved from α-methylthio-*p*-cyanoacetophenone to 1,2-diphenylethanone under chemical equilibrium. This method is applied to cyclic α-phenyl ketones with acidic α-protons. The reaction of 2-phenylthio-4-(*t*-butyl) cyclohexanone and α-methylthio-*p*-chloroacetophenone gave a product with an axial methylthio group, and the cleavage of the phenylthio group was slow under these conditions [43].

2-Methylthio-1,2-diphenylethanone is effective in the α-methylthiolation reactions of ketones with less acidic α-protons compared with α-methylthio-*p*-cyanoacetophenone [44]. Reaction of 2-benzylcyclohexanone and 2-methylthio-1,2-diphenylethanone in the presence of RhH(PPh_3_)_4_ (4 mol%), dppe (8 mol%), and dimethyl disulfide (12 mol%) in refluxing THF for 3 h gave 2-benzyl-2-methylthiocyclohexanone in 47% yield. The regioselectivity indicated the important role of the enol form of 2-methylcyclohexanone. The method is also applied to α-methylthiolation of aldehydes. In these α-thiolation reactions, disulfides alone did not give satisfactory results, and α-thioketones were employed as the donors. A favorable chemical equilibrium may be involved in forming ketones from α-thioketones rather than forming thiols from disulfides. Possible roles of dimethyl disulfide are the generation of reactive rhodium species with sulfur ligands and/or the transient formation of α-methylthio ketones.

Phenylthiolation reactions of aromatic hydrogen atoms in benzo-fused heteroaromatic compounds were conducted using α-phenylthioisobutyrophenone, which provided higher yields of the products when compared with 2-methylthio-1,2-diphenylethanone [45]. This result may be due to the thermodynamically favorable nature of chemical equilibrium to form a ketone with a less acidic α-proton. 1,3-Benzothiazole and α-methylthioisobutyrophenone were reacted in chlorobenzene reflux for 3 h in the presence of RhH(PPh_3_)_4_ (4 mol%) and dppe (8 mol%), which provided 2-phenylthio-1,3-benzothiazole in 92% yield. The methylthiolation reaction of benzothiazole provided α-methythioisobutyrophenone in the presence of dimethyl disulfide, albeit in lower yields. 

**Figure 6:**
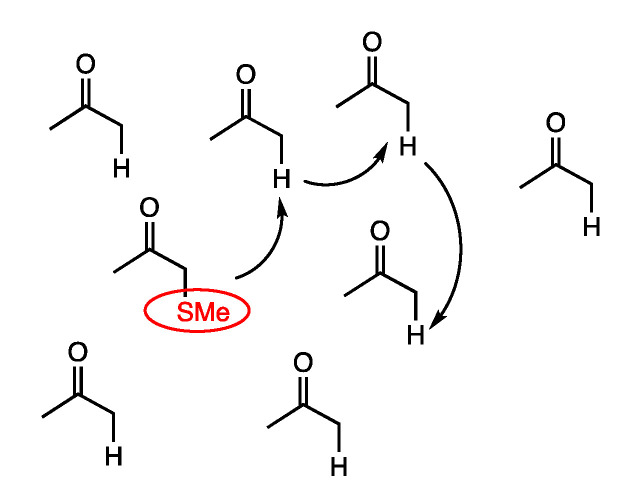
Rhodium-catalyzed hopping of methylthio groups among ketones at α-positions.

These results indicate that methylthio groups can hop between α-protons of ketones under rhodium catalysis (Figure 6). This is analogous to the α-proton exchange reactions between ketones under acidic or basic conditions.

### 3.5. Substitution Reactions of Aromatic Fluorides

**Scheme 12:**
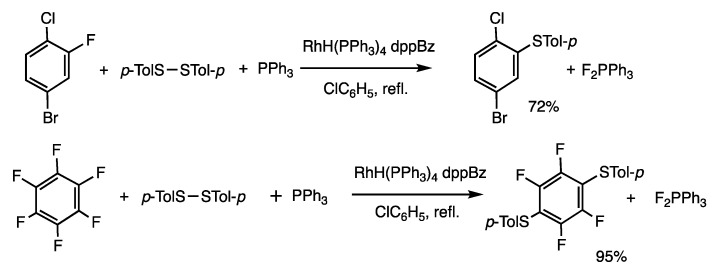
Substitution reaction of aromatic fluorides.

Organofluorine compounds are highly reactive under rhodium catalysis, as noted in the reaction of acyl fluorides to form thioesters (Scheme 5). This behavior may be due to the high reactivity of rhodium fluoride intermediates, which is consistent with the notably high reactivity of RhF(PPh_3_)_3_ [46]. Accordingly, C-F bonds in fluorobenzenes were converted to C-S bonds via reaction with disulfides (Scheme 12). When 1-bromo-4-chloro-3-fluorobenzene was reacted with bis(*p*-tolyl) disulfide and triphenylphosphine in the presence of RhH(PPh_3_)_4_ (0.25 mol%) and 1,2-(diphenylphosphino) benzene (dppBz) (0.5 mol%) under chlorobenzene reflux for 3 h, 5-bromo-2-chlorophenyl *p*-tolyl sulfide was obtained in 72% yield [47]. The reaction proceeded selectively at the fluoride atom without affecting chloride and bromide atoms. Triphenylphosphine was employed to trap fluorides by forming phosphine difluoride, which resulted in an exothermic reaction. The reaction of perfluorobenzenes showed notable selectivity in the substitution reaction, and thiolation occurred at the *p*-positions, which was named the *p*-difluoride rule. The reaction of hexafluorobenzene and bis(*p*-tolyl) disulfide gave 1,4-bis(*p*-tolyl)-2,3,5,6-tetrafluorobenzene, in which no monothiolated product was detected.

### 3.6. Substitution Reactions of Organophosphorus Compounds

**Scheme 13:**
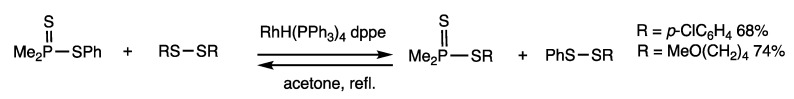
Organothio exchange reaction of dithiophosphinates.

Organophosphorus compounds efficiently react with disulfides in rhodium-catalyzed substitution reactions, owing to the strong bonding of sulfur and phosphorus atoms. Cleavage of the P-S bond was determined by organothio exchange reactions of dithiophosphinates and disulfides (Scheme 13) [48]. When phenyl dimethyldithiophosphinate and bis(4-methoxybutyl) disulfide were reacted in the presence of RhH(PPh_3_)_4_ (2 mol%) and dppe (4 mol%) under acetone reflux for 0.5 h, the corresponding dithiophosphinate was obtained in 74% yield.

**Scheme 14:**
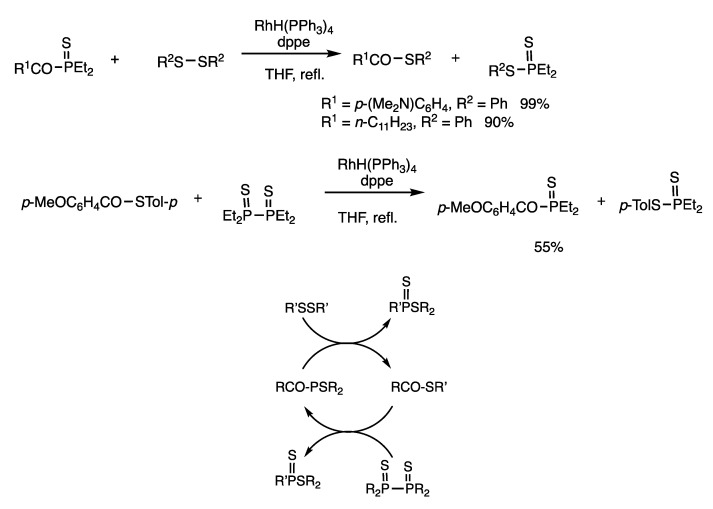
Interconversion reactions between acylphosphine sulfides and thioesters.

Reactions of acylphosphine sulfides and disulfides provided dithiophosphinates and thioesters, which involved the formation of C-S and P-S bonds from C-P and S-S bonds (Scheme 14) [49]. When diethyl(*p*-dimethylaminobenzoyl) phosphine sulfide was reacted with di(undecyl) disulfide in the presence of RhH(PPh_3_)_4_ (2 mol%) and dppe (4 mol%) in refluxing THF for 3 h, the corresponding thioester was obtained in 90% yield, along with dithiophosphonate.

Thioesters were converted to acylphosphine sulfides by reacting with diphosphine sulfides (Scheme 14) [49]. When S-(*p*-tolyl) *p*-methoxybenzothioate and tetraethyldiphosphine disulfides were reacted in refluxing chlorobenzene for 6 h in the presence of RhH(PPh_3_)_4_ (5 mol%) and 1,2-(diethylphosphino) ethane (depe) (10 mol%), acylphosphine sulfide was obtained in 55% yield. Thus, interconversion reactions in an open system occur between thioesters and acylphosphines in the presence of appropriate reagents, which are catalyzed by rhodium complexes; i.e., organosulfur compounds and organophosphorus compounds may be interconverted.

**Scheme 15:**
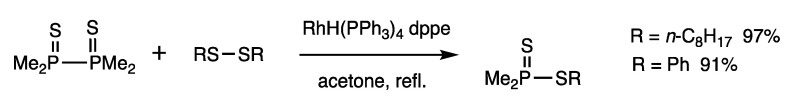
Exchange reaction of diphosphine disulfides and disulfides.

On the basis of analysis of interconversion reactions in open systems, it was suggested that the treatment of diphosphine disulfides and disulfides provides dithiophosphinates via the exchange of P-P bonds and S-S bonds (Figure 4) [48]. When dioctyl disulfide and tetramethyldiphosphine disulfide were reacted under acetone reflux for 0.5 h in the presence of RhH(PPh_3_)_4_ (1.5 mol%) and dppe (3 mol%), thiophosphinate was obtained in 97% yield (Scheme 15). The reaction is irreversible, owing to the formation of strong P-S bonds from weak P-P bonds. The reaction is also applicable to diselenides.

**Scheme 16:**
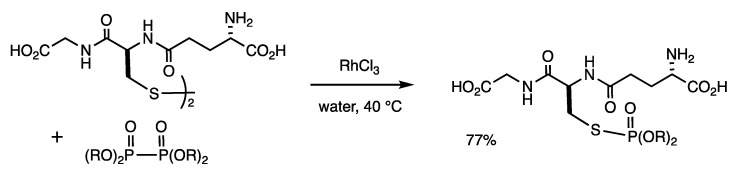
Exchange reaction of diphosphine disulfides and disulfides in water.

The reaction of hydrophilic disulfides and diphosphonates, which are also water-soluble, proceeds in water in the presence of RhCl_3_ (Scheme 16) [50]. Glutathione disulfide was reacted with tetramethyl diphosphonate in water at 40 °C for 36 h in the presence of RhCl_3_ (10 mol%), and glutathione phosphonate was obtained in 77% yield.

**Scheme 17:**
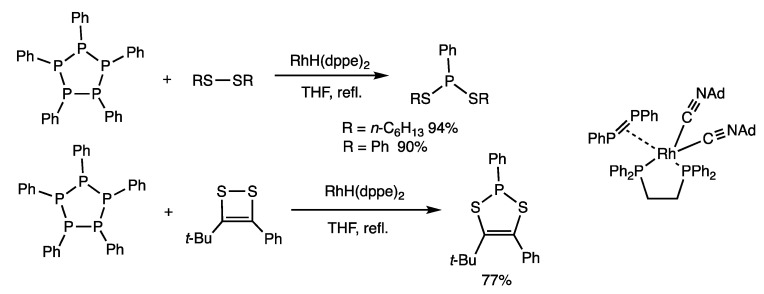
Exchange reaction of cyclopentaphosphine and disulfides.

Polyphosphines are compounds containing phosphorus atoms with two P-P bonds and one organic group, and they exhibit reactivities different from those of diphosphines, which contain phosphorus atoms with one P-P bond and two organic groups. Polyphosphines undergo substitution reactions with disulfides, which involve the exchange of P-P and S-S bonds (Scheme 17) [51]. Pentaphenylcyclopentaphosphine was reacted with dihexyl disulfide in the presence of RhH(dppe)_2_ (5 mol%) in THF reflux for 15 min, which produced dihexyl phenylphosphonodithionite in 94% yield. When cyclic disulfides are employed, novel heterocyclic phosphonodithinites are obtained. The intermediate formation of a diphosphene–rhodium complex was shown by spectroscopic and MS analyses.

**Scheme 18:**
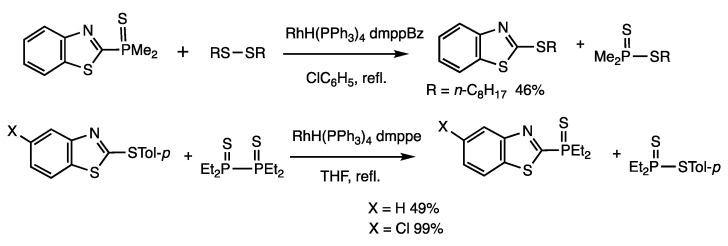

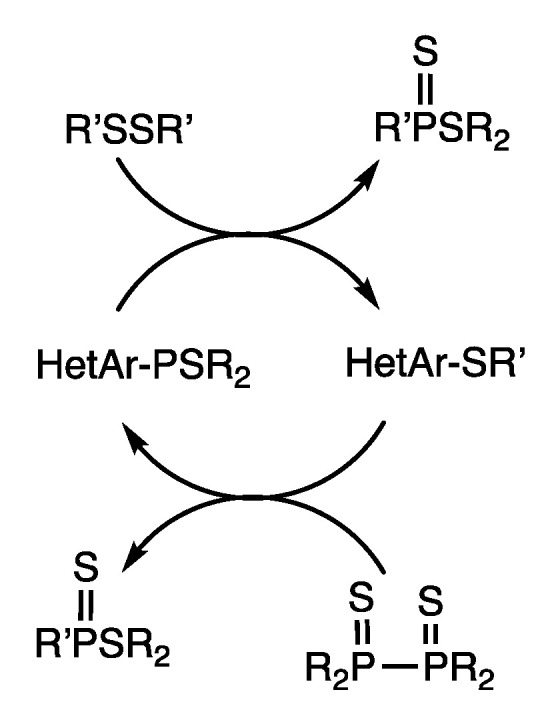
Interconversion reactions of heterocyclic phosphine sulfides and heterocyclic sulfides.

A rhodium catalyst promoted the cleavage of C-P bonds in heteroarylphosphine sulfides and exchange with disulfides (Scheme 18) [52]. When (2-benzothiazolyl) dimethylphosphine sulfide was reacted with dioctyl disulfide in the presence of RhH(PPh_3_)_4_ (10 mol%) and 1,2-bis(dimethylphosphino)benzene (dmppBz) (20 mol%) in refluxing chlorobenzene for 3 h, 2-(octylthio) benzothiazole was obtained in 46% yield. 

The reverse reaction converts heteroaryl sulfides to heteroarylphosphine sulfides in the presence of diphosphine disulfides (Scheme 18) [52,53]. When 2-(*p*-tolylthio)-1,3-benzothiazole and tetraethyldiphosphine disulfides were reacted in refluxing chlorobenzene for 6 h in the presence of RhH(PPh_3_)_4_ (5 mol%) and 1,2-(diethylphosphino) ethane (depe) (10 mol%), 2-(diethylphosphino)-1,3-benzothiazole sulfide was obtained in 49% yield. This is another example of interconversion reactions in an open system, in which organophosphorus and organosulfur compounds are interconverted.

## 4. Rhodium-Catalyzed Insertion Reactions Using Disulfides

**Scheme 19:**
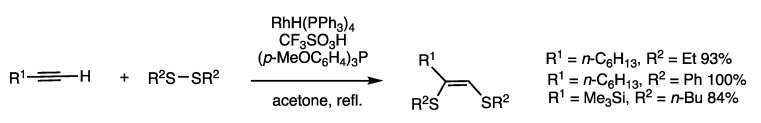
Insertion reaction of disulfides and 1-alkynes.

Insertion reactions of disulfides and alkynes/alkenes are generally exothermic, involving the conversion of high-energy unsaturated compounds to low-energy less unsaturated compounds. Previously reported palladium-catalyzed insertion reactions of disulfides with alkynes in general employed diaryl disulfides [21,23]. Rhodium-catalyzed insertion of 1-akynes may be applied to aliphatic disulfides, which are less reactive than aromatic disulfides (Scheme 19) [54]. 

When 1-octyne and diethyl disulfide were reacted in refluxing acetone for 10 h in the presence of RhH(PPh_3_)_4_ (4 mol%), trifluoromethanesulfonic acid (4 mol%), and (*p*-MeOC_6_H_4_)_3_P (14 mol%), 1,2-bis(alkylthio)-1-alkene with the Z-configuration was obtained in 93% yield. The proposed mechanism involves the oxidative addition of low-valent rhodium and a disulfide, followed by the transfer of two organothio groups to 1-alkyne.

**Scheme 20:**
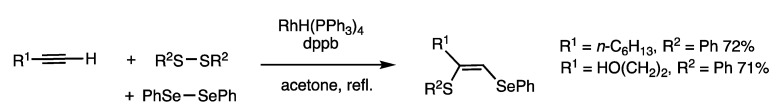
Insertion reaction of disulfides/diselenides and 1-alkynes.

A mixture of a disulfide and a diselenide predominantly provides 1-seleno-2-thioalkene, which is accompanied by minor amounts of other insertion products (Scheme 20) [55]. When 1-octyne, diphenyl disulfide, and diphenyl diselenide were reacted in refluxing acetone for 4 h in the presence of RhH(PPh_3_)_4_ (5 mol%) and 1,4-(diphenylphosphino) butane (dppb) (10 mol%), 2-butylthio-1-butylseleno-1-octene was obtained in 72% yield. The reaction is also applicable to dialkyl disulfides/diselenides. The reaction is under chemical equilibrium of disulfide/diselenide exchange, in which selenosulfides are selectively transferred to 1-alkynes.

**Scheme 21:**
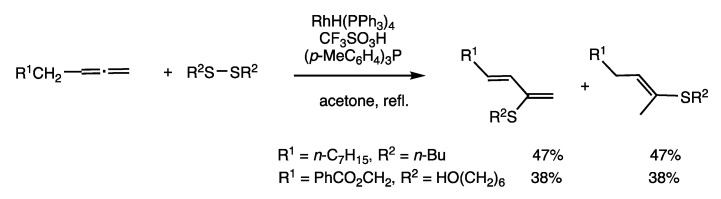
Insertion reaction of allenes and disulfides.

The reaction of allene in place of 1-alkyne provides a different variety of products (Scheme 21) [56]. When 1,2-octadiene and dibutyl disulfide were reacted in refluxing acetone for 2 h in the presence of RhH(PPh_3_)_4_ (3 mol%), trifluoromethanesulfonic acid (3 mol%), and (*p*-tol)_3_P (12 mol%), 2-(butylthio)-1,3-octadiene and (*E*)-2-(butylthio)-2-octene were both obtained in 47% yields. Two organothio groups are transferred to two allene molecules with concomitant hydride transfer. This reaction is also applicable to diselenides.

**Scheme 22:**
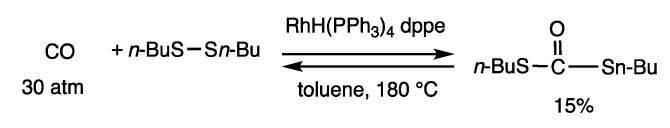
Insertion reaction of carbon monoxide and disulfide.

The α-insertion reaction of carbon monoxide with disulfide occurred to provide dithiocarbonate (Scheme 22) [36]. When dibutyl disulfide was treated with carbon monoxide at 30 atm in toluene at 180 °C for 24 h in the presence of RhH(PPh_3_)_4_ (10 mol%) and dppe (20 mol%), dibutyl *S*, *S*’-dithiocarbonate was obtained in 15% yield. This reaction is under equilibrium and favors disulfide and carbon monoxide at ambient pressure as well as at 30 atm. Rhodium-catalyzed α-insertion reactions of carbenoids and disulfides have been reported [57,58].

## 5. Rhodium-Catalyzed Reduction/Oxidation Reactions of Disulfides

**Scheme 23:**
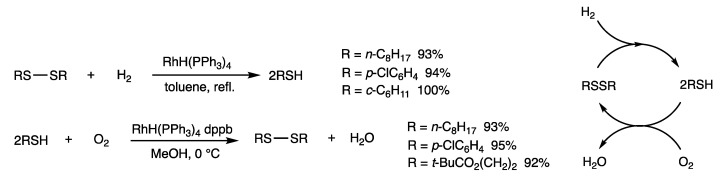
Interconversion reactions of disulfides and thiols.

Interconversion of a disulfide and two thiols is an important chemical reaction in organosulfur chemistry. Various methods have been reported for the reduction of disulfides to thiols using different reducing reagents. Hydrogenation may be the most convenient in terms of availability of reducing reagent and simple operations, which is also achieved by rhodium catalysis (Scheme 23). When di(octyl) disulfide was treated with hydrogen at 1 atm in the presence of RhH(PPh_3_)_4_ (0.5 mol%) in refluxing toluene for 0.5 h, 1-octanethiol was obtained in 90% yield [59]. Care to deactivate a rhodium complex is critical during quenching of the reaction because thiols are rapidly converted to disulfide under air in the presence of the rhodium complex. Metal-catalyzed hydrogenolysis of disulfides to thiols has been rare, which may be because of catalyst poisoning by thiols.

The reverse reaction, oxidation of thiols to disulfides, is also catalyzed by the rhodium complex using oxygen as the oxidation reagent (Scheme 23) [59]. Treatment with 1-octanethiol under oxygen at 1 atm in methanol at 0 °C for 1 h in the presence of RhH(PPh_3_)_4_ (0.1 mol%) and 1,4-bis(diphenylphosphino)butane (dppb) (0.2 mol%) provided dioctyl disulfide in 93% yield.

These interconversion reactions in open systems can be a convenient method for the development of molecular switching functions, which employ hydrogen as the reducing reagent and oxygen as the oxidizing reagent.

## 6. Rhodium-Catalyzed Reactions of Sulfur

Sulfur is a readily available source of organosulfur compounds in organic synthesis. Conventional methods using sulfur generally employ the formation of thiyl radicals, which are generated by heating above the melting point of sulfur (115 °C) [30]. Several previously reported metal-catalyzed methods employ high temperatures, which likely involve radical- and metal-catalyzed mechanisms and accordingly are not easy to analyze and control. To secure the effect of metal catalysis, we conducted rhodium-catalyzed reactions of sulfur well below the melting point. The reactivity of sulfur can be different from disulfide, and Rh-S-S intermediates formed from sulfur can exhibit different reactivities from Rh-S-C intermediates formed from disulfides because of the presence of the adjacent weak S-S bonds in the latter. 

**Scheme 24:**
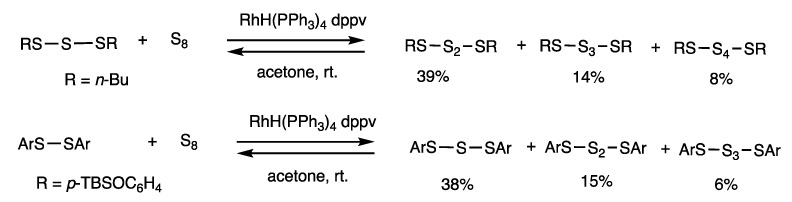
Insertion reaction of sulfur and disulfides.

Introduction of sulfur atoms between S-S bonds in disulfides provides polysulfides, and such reactions occur above the melting point and involve radical mechanisms. Rhodium-catalyzed reactions proceed at much lower temperatures [60]. In the presence of RhH(PPh_3_)_4_ (2.5 mol%) and 1,2-bis(diphenylphosphino)ethene (dppv) (5 mol%), dibutyl trisulfide and sulfur were reacted in acetone at room temperature for 5 min, and dibutyl tetrasulfide, pentasulfide, and hexasulfide were obtained in 39, 14, and 8% yields, respectively, accompanied by higher homologs (Scheme 24). The reaction occurred rapidly and provided a mixture of polysulfides. Aliphatic disulfides were not reactive under these conditions. In contrast, diaryl disulfides effectively reacted with sulfur to provide polysulfides, including trisulfides, tetrasulfides, and pentasulfides.

**Scheme 25:**
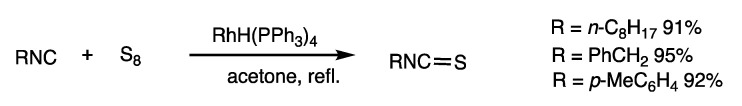
Insertion reaction of sulfur and thioisonitriles.

Thioisonitriles were synthesized by sulfuration of isonitriles under rhodium-catalyzed conditions, which resulted in the 1,1-insertion of sulfur at the nitrogen atom of the C=N bond (Scheme 25) [61]. The reaction of cyclohexylisonitrile and sulfur under acetone reflux for 2 h in the presence of RhH(PPh_3_)_4_ (1 mol%) provided thioisonitrile in 91% yield in 2 h. The reaction is faster than the known molybdenum method developed by Bargon [62]. Trisulfides and tetrasulfides can also react under rhodium catalysis. An induction period was observed with an initially slow reaction for 40 min followed by a rapid reaction completed in 100 min. The phenomenon was ascribed to the slow formation of active sulfur species because preheating sulfur in acetone for 1.5 h eliminated the induction period.

**Scheme 26:**
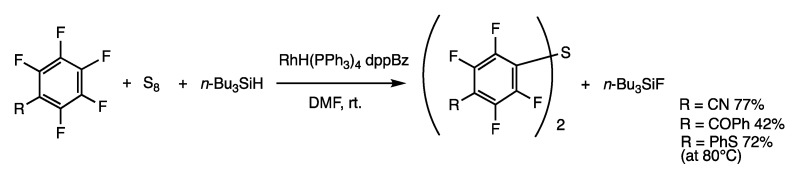
Insertion reaction of sulfur and aryl fluorides.

Symmetric diaryl sulfides are synthesized from perfluorobenzene and sulfur involving C-F bond activation, which showed reactivity and the *p*-difluoride rule similar to disulfides (Scheme 26) [63]. Treatment of 4-phenylthiopentafluorobenzene, sulfur, and tributylsilane in DMF at room temperature in the presence of RhH(PPh_3_)_4_ (5 mol%) and dppBz (10 mol%) provided bis(4-cyano-2,3-5,6-tetrafluorophenyl) sulfides in 77% yield. Fluorides were trapped by trialkylsilane giving silyl fluoride. Aromatic fluorides in place of polyfluorobenzenes also reacted, provided that electron-withdrawing groups were attached. Trisulfide and tetrasulfide may also be used as the sulfur source, but they exhibit reactivities different from those of sulfur.

**Scheme 27:**
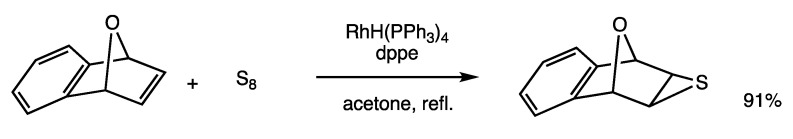
Insertion reaction of sulfur and alkene.

Thiiranes are three-membered heterocyclic ring compounds containing a sulfur atom, and the insertion reaction of a sulfur atom at the alkene C=C bond is a convenient method for their synthesis [64]. The reaction may be efficiently conducted by rhodium catalysis (Scheme 27) [65]. When 7-oxabenzonorbornene, sulfur, and *p*-tolylacetylene were reacted in the presence of RhH(PPh_3_)_4_ (5 mol%) and dppe (10 mol%) in refluxing acetone for 3 h, exo-thiirane was obtained in 91% yield. The acetylene added stabilizes the rhodium intermediates. The isolated RhH(dppe)_2_ also exhibited catalytic activity. The reaction is applicable to reactive alkenes, including bicyclo[2.2.1]heptenes, allenes, and (Z)-cyclooctene.

**Scheme 28:**
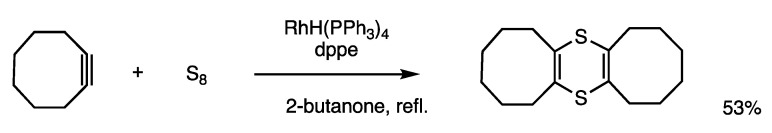
Insertion reaction of sulfur and alkyne.

1,4-Dithiine is a nonaromatic six-membered ring heterocyclic compound with a nonplanar structure. Dithiines are synthesized by the rhodium-catalyzed reaction of reactive alkynes and sulfur, which involves an insertion reaction of an alkyne between a S-S bond of sulfur (Scheme 28) [66]. The reaction of cyclooctyne and sulfur in refluxing 2-butanone for 3 h in the presence of RhH(PPh_3_)_4_ (5 mol%) and dppe (10 mol%) provided tricyclic dithiine in 53% yield. When acetylenedicarboxylate was added, unsymmetric dithiine was selectively obtained without the formation of a symmetric dithiine.

Rhodium-catalyzed synthesis of organosulfur compounds using sulfur has high potential in organic synthesis because it proceeds well below the melting point of sulfur. An interesting consideration is their application to less reactive substrates such as unstrained alkenes and alkynes, which may be developed employing thermodynamically favorable reaction systems.

## 7. Reversible Nature of Rhodium-Catalyzed C-S Bond Formation

**Scheme 29:**
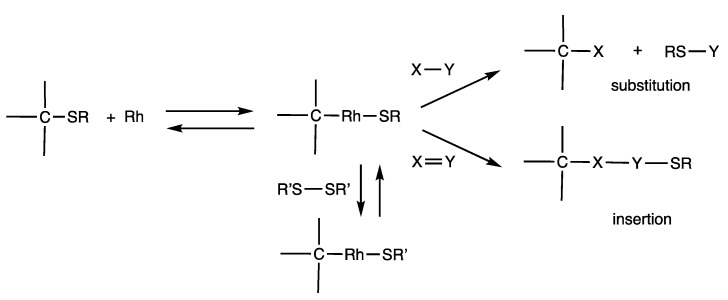
Reversible nature of rhodium-catalyzed substitution reactions.

Rhodium-catalyzed reactions of disulfides provide various organosulfur compounds by the cleavage of RS-SR bonds and the formation of C-SR bonds, and they involve C-Rh-SR intermediates (Scheme 29). Because C-SR bond formation reactions are often reversible, C-Rh-SR intermediates can also be formed from products with C-SR bonds. The C-Rh-SR intermediates can undergo various substitution and insertion reactions to provide organic compounds. Insertion reactions of C-Rh-SR intermediates with X=Y bond compounds provide C-X-Y-SR compounds, which are generally irreversible reactions. Substitution reactions with compounds containing X-Y bonds provide novel C-X bond compounds accompanied by compounds containing RS-Y bonds, and these reactions are often reversible. This is a substitution reaction of C-SR compounds to C-X compounds, in which the role of the SR group is that of a formal leaving group. The RS-Y bond compounds are organosulfur compounds that are easy to recover and use and can be converted to other organosulfur compounds. When C-Rh-SR intermediates undergo an organothio exchange with another disulfide R’S–SR’, the resulting C-Rh-SR’ intermediates also undergo various reactions. This is a chemical reaction network, which provides diverse organosulfur compounds involving chemical equilibrium and interconversion reactions. In this section, we describe the reactivities of organosulfur compounds synthesized by rhodium-catalyzed reactions using disulfides. 

In organic synthesis, substitution reactions are generally conducted using organohalogen compounds; in this article, we describe the use of organosulfur compounds for such purpose. Halogens and organothio groups are leaving groups, which can exhibit different properties of reactions. For example, organohalogens are involved in irreversible reactions, and organosulfurs can be involved in reversible reactions, which provide diverse organosulfur compounds by slight changes in catalysts and/or reaction conditions. The organohalogen reactions form metal halides, which are not easy to recover and reuse; the organosulfur reactions provide organosulfur compounds for coproducts, which can be used for various purposes.

### 7.1. Reactions of 1-Thioalkynes

**Scheme 30:**
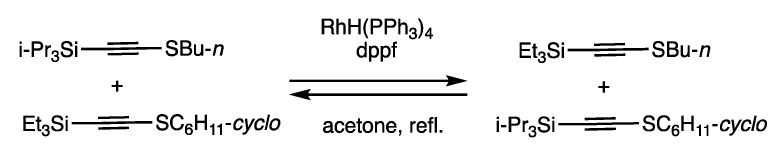
Organothio exchange reaction between 1-thioalkynes.

As shown previously, the C-S bonds in 1-thioalkynes are readily activated by rhodium catalysis (Scheme 7 and Scheme 8) [39], and then 1-thioalkynes can be used for substitution and insertion reactions. Organothio groups can be exchanged between different 1-thioalkynes, which confirmed the reversible cleavage of C-S bonds (Scheme 30) [67]. Two 1-thioalkynes in refluxing acetone in the presence of RhH(PPh_3_)_4_ (1 mol%) and bidentate dppf (2 mol%) provided a mixture containing comparable amounts of four possible 1-thioalkynes under chemical equilibrium. This is referred to as C-S/C-S to C-S/C-S metathesis.

**Scheme 31:**
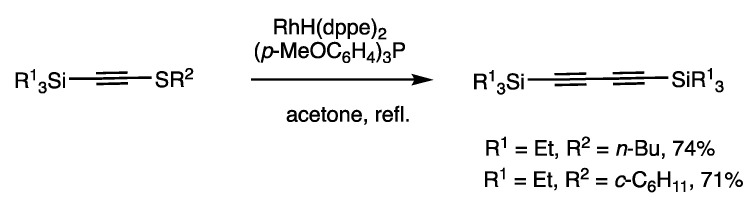
Oxidative coupling reaction of 1-thioalkynes.

The ligand effect was substantial in the reactions of 1-thioalkynes, and the coupling reaction of 1-thioalkynes occurred in the presence of a monodentate ligand (Scheme 31). The reaction of 1-(triethylsilyl)-2-butylthioalkyne in refluxing acetone for 2 h in the presence of RhH(PPh_3_)_4_ (1 mol%) and a monodentate ligand (*p*-MeOC_6_H_4_)_3_P (3 mol%) gave 1,3-diyne in 74% yield and disulfide. This oxidative coupling reaction is referred to as C-S/C-S to C-C/S-S metathesis, which is in contrast to the above C-S/C-S to C-S/C-S metathesis (Scheme 30). 

**Scheme 32:**
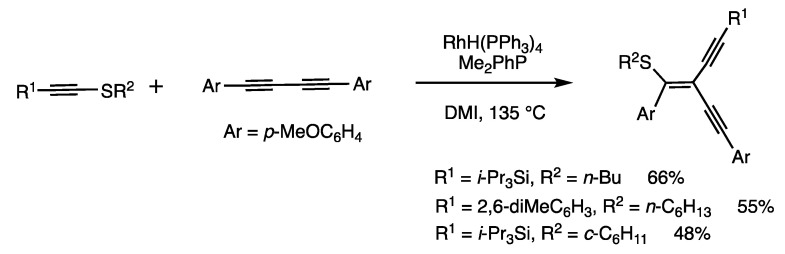
Insertion reaction of 1-thioalkynes and alkynes.

The insertion reaction of 1-thioalkynes and unsaturated compounds provides novel organosulfur compounds, and 1,3-butadiyne is used as a substrate (Scheme 32) [68]. The reaction of 1-butylthio-2-triethylsilyletyne and 1,4-(*p*-methoxyphenyl)-1,3-butadiyne in *N*, *N*-dimethylimodazolinone (DMI) at 135 °C for 6 h in the presence of RhH(PPh_3_)_4_ (5 mol%) and Me_2_PhP (10 mol%) gave 1-butylthio-2-ethynyl-1,3-buten-yne in 66% yield, which is a highly unsaturated organosulfur compound. 

**Scheme 33:**
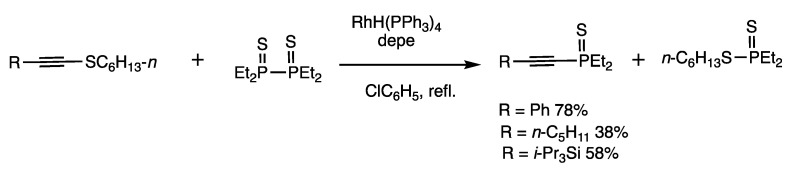
Exchange reaction of diphosphine disulfide and 1-thioalkynes.

The conversion of 1-thioalkynes to organophosphorus compounds proceeds under rhodium catalysis by reacting with diphosphine disulfides (Scheme 33) [53]. When 1-hexylthio-2-(2,4,6-trimethylphenyl) acetylene and tetramethyldiphosphine disulfide were reacted in refluxing chlorobenzene for 12 h in the presence of RhH(PPh_3_)_4_ (2 mol%) and 1,2-(diethylphosphino) ethane (depe) (4 mol%), 1-alkynylphosphine sulfide was obtained in 88% yield. 1-Thioalkynes under rhodium catalysis exhibit different reactivities from 1-alkynes and 1-haloalkynes; for example, no base or organometallic reagents are used in these reactions.

### 7.2. Reactions of Thioesters

Thioesters are reactive substrates in rhodium-catalyzed reactions and undergo various chemical transformations that are not observed with acyl halides. The reversible cleavage of C-S bonds was determined in studies of reactions of thioesters and disulfides (Scheme 4). Thioesters were applied to acylation reactions, forming C-S, C-P, C-N, and C-C bonds.

**Scheme 34:**
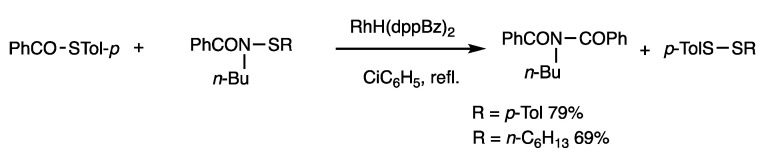
Oxidative coupling reaction of thioester and *N*-thioamides.

Thioesters react with *N*-organothioacylamides to provide acylimides with concomitant formation of disulfides, which is a novel acylation reaction at an amide nitrogen atom (Scheme 34) [69]. *S*-(*p*-Tolyl) benzothioate and *N*-(*p*-tolylthio)-*N*-butylbenzamide were reacted in refluxing chlorobenzene for 6 h in the presence of RhH(dppBz)_2_ (5 mol%), and *N*-benzoyl-*N*-butylbenzamide was obtained in 79% yield. This is a novel metal-catalyzed coupling reaction involving N-C bond formation with cleavage of C-S and S-N bonds in two organosulfur compounds followed by oxidative elimination of disulfides.

**Scheme 35:**
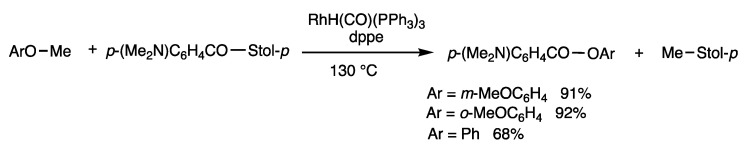
Substitution reaction of thioester and aryl methyl ethers.

Aryl methyl ethers are cleaved with thioesters under rhodium-catalyzed conditions (Scheme 35) [70]. The reaction of *m*-dimethoxybenzene and *S*-(*p*-tolyl) *p*-dimethylaminobenzothioate at 130 °C for 12 h without a solvent in the presence of RhH(CO)(PPh_3_)_3_ (8 mol%) and dppe (16 mol%) provided *m*-methoxyphenyl benzoate in 91% yield along with *p*-tolyl methyl sulfide. This cleavage reaction of an aryl methyl ether to provide an aryl ester proceeds under neutral conditions without using strong nucleophiles or Lewis acids.

**Scheme 36:**
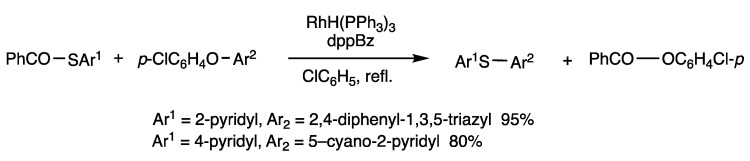
Substitution reaction of thioester and diaryl ethers.

Heteroaryl aryl ethers are reacted with aryl thioesters to provide unsymmetric bis(heteroaryl) sulfides (Scheme 36) [71]. *S*-(3-Pyridyl) benzothioate and 2-benzothiazolyl 4-chlorophenyl ether were reacted in refluxing chlorobenzene for 5 h in the presence of RhH(PPh_3_)_4_ (5 mol%) and dppBz (10 mol%) and provided 3-pyridyl 2-benzothiazolyl sulfide in 95% yield. The other reaction pathway to form an aryl sulfide and a heteroaryl ester did not proceed. Very few unsymmetric bis(heteroaryl) sulfides were previously known, and this method provides novel multiple heteroatom compounds, some of which show interesting biological activities.

**Scheme 37:**
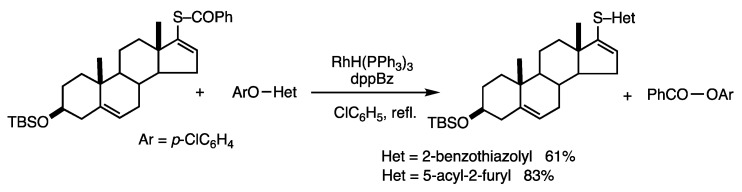
Substitution reaction of thioester and aryl heteroaryl ethers.

This reaction is applicable to the synthesis of sulfides with aliphatic cyclic groups and heteroaryl groups [72]. Specifically, the reaction of a steroidal benzothioate provided steroidal heteroaryl sulfides (Scheme 37). This is an interesting synthesis of alkyl aryl sulfides that does not require the use of bases.

Rhodium catalysts cleave C-C bonds of benzyl ketones, which then react with thioesters and esters [73]. When S-methyl benzothioate was reacted with 2-(2-thienyl)-1-(*p*-cyanophenyl)-1-ethanone in DMI at 150 °C for 12 h in the presence of RhH(CO)(PPh_3_)_3_ (10 mol%) and dppBz (20 mol%), a benzyl-exchanged ketone was obtained in 76% yield. The benzyl group was transferred between different thioesters by the cleavage of a C-C bond under chemical equilibrium. The other pathway of the bond exchange reaction providing benzyl sulfide and 1,2-diketones did not occur, which may be due to thermodynamic reasons involving the substrates and products. The use of an aryl ester in place of the thioester also gives benzyl-exchanged products. 

These results indicate that the rhodium complex catalytically cleaves C-C bonds of unstrained benzyl ketones. The reaction of aryl ethers in place of thioesters/esters provided a route to form diarylmethanes [74]. The reaction of 2-(*p*-chlorophenoxy)-5-acetylfuran and 2-(1,3-benzoxazolyl)-1-phenyl-1-ethanone in refluxing chlorobenzene for 6 h in the presence of RhH(PPh_3_)_4_ (10 mol%) and dppBz (20 mol%) provided (1,3-benzoxazolyl) (5-acetylfuryl) methane in 53% yield along with phenyl benzoate. The synthesis is applicable to the production of various unsymmetric bis(heteroaryl)methanes that were previously not known. In addition, diarylmethanes are obtained from neutral compounds by the cleavage and formation of C-C bonds without using bases or organometallic reagents. 

**Scheme 38:**
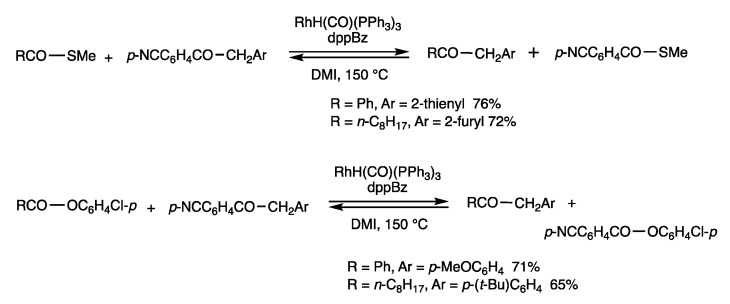
Substitution reaction of thioester/esters and arylmethyl ketones.

**Scheme 39:**
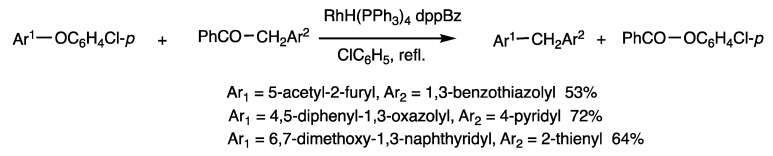
Substitution reaction of diaryl ethers and arylmethyl ketones.

**Scheme 40:**
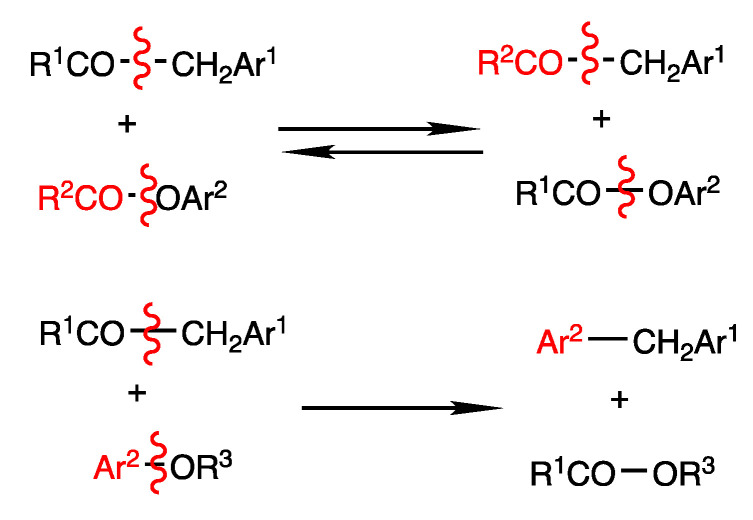
Comparison of substitution reactions of arylmethyl ketones and aryl esters/aryl ethers.

The reactions in Scheme 38 and Scheme 39 are notable examples of rhodium-catalyzed cleavage of C-C bonds in benzyl ketones, in which different types of reactions proceed depending on the substrates used (Scheme 40). The benzyl exchange reaction of esters and thioesters proceeds by CO-OAr^2^ bond cleavage, which is under chemical equilibrium; the substitution reaction of ethers provided diarylmethane and esters by O-Ar^2^ bond cleavage. 

**Scheme 41:**
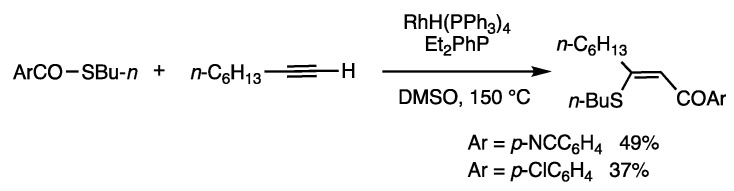
Insertion reaction of thioesters and 1-alkyne.

Rhodium-catalyzed insertion reactions of thioesters occur with 1-alkynes (Scheme 41) [49]. When *S*-butyl *p*-cyanobenzothioate was reacted with 1-decyne in DMSO at 100 °C for 12 h in the presence of RhH(PPh_3_)_4_ (5 mol%) and Et_2_PhP (15 mol%), a conjugated enone was obtained in 49% yield. The benzoyl group was attached at the terminal carbon atom of the 1-alkyne and the organothio group at the internal carbon atom.

**Scheme 42:**
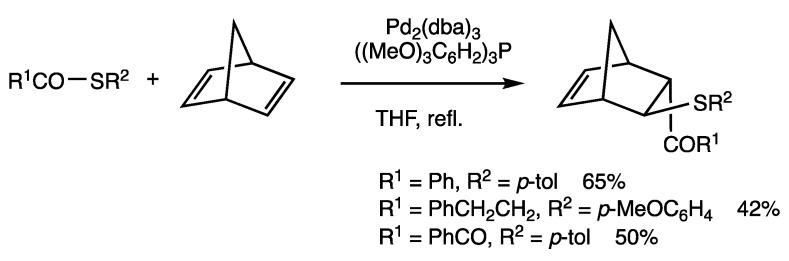
Insertion reaction of thioesters and alkene.

Insertion reactions of thioesters occur with strained alkenes (Scheme 42) [75]. The reaction of *S*-(*p*-tolyl) benzothioate and norbornadiene under THF reflux for 6 h in the presence of Pd_2_(dba)_3_ (dba = dibenzylideneacetone) (5 mol%) and (2,4,6-(MeO)_3_C_6_H_2_)_3_P (20 mol%) gave the acylated adduct with trans configuration, in which an acyl group is attached in the endo position. The initial bond formation is considered to involve acylation, which is followed by thiolation.

### 7.3. Reactions of α-Thioketone

**Scheme 43:**
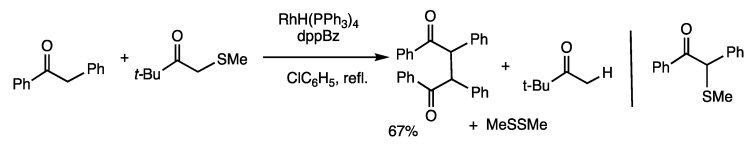
Oxidative coupling reaction of ketones.

The C-S bonds in α-thioketones are activated by rhodium catalysts to form oxidative addition intermediates with S-Rh-C subunits, which react with C-H bonds in organic compounds (Scheme 11) [43,44]. The activation of α-thioketones can be used for the coupling reaction to form C-C bonds at the ketone α-position (Scheme 43) [76]. The reaction of 1,2-diphenyl-1-ethanone and methylthiomethyl *t*-butyl ketone in refluxing chlorobenzene for 6 h in the presence of RhH(PPh_3_)_4_ (10 mol%) and dppBz (20 mol%) gave a dimeric ketone at the α-position in 67% yield. The reaction is considered to involve the initial transfer of the methylthio group to 1,2-diphenyl-1-ethanone followed by coupling to form diketone along with dimethyl disulfide. The mechanism was determined from the reaction between α-methylthiolated 1,2-diphenyl-1-ethanone and 1,2-diphenyl-1-ethanone to provide the coupling product. It should be noted that oxidative dimerization of ketones can be conducted under rhodium catalysis using α-thioketones as oxidation reagents.

### 7.4. Reactions of Aryl/Heteroaryl Sulfides

**Scheme 44:**
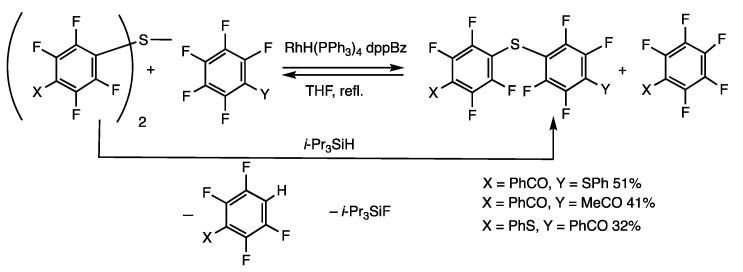
Exchange reaction of diaryl sulfides and aryl fluorides.

The rhodium-catalyzed C-S bond cleavage of aryl sulfides provided other aryl sulfides by the exchange of arylthio groups [77]. Polyfluorophenyl and heteroaryl derivatives are highly reactive substrates for the cleavage and formation of C-S bonds, and symmetric diaryl sulfides can be converted into unsymmetric diaryl sulfides (Scheme 44). Perfluorinated bis(*p*-benzoylphenyl) sulfide, (phenylthiol)pentafluorobenzene, and triisopropylsilane were reacted in the presence of RhH(PPh_3_)_4_ (5 mol%) and dppBz (10 mol%) in refluxing THF for 6 h and provided unsymmetric diaryl sulfide in 51% yield. Silane was added to trap fluoride by the formation of silyl fluoride, which resulted in an exothermic reaction. The reaction is under chemical equilibrium without silane, and the unsymmetric diaryl sulfide can also undergo the aryl exchange reaction. Combined with the synthesis of symmetric diaryl sulfides from polyfluorobenzene and sulfur (Scheme 26), various unsymmetric diaryl sulfides can be synthesized using sulfur.

**Scheme 45:**
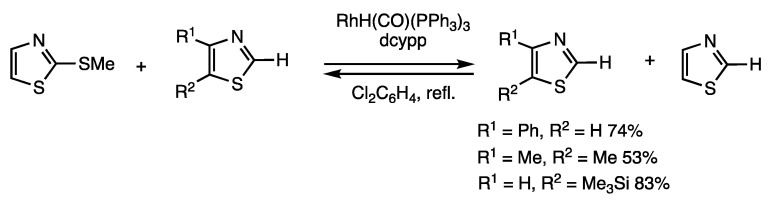
Organothio exchange reaction of thiazoles.

Benzo-fused heteroarenes are thiolated under rhodium-catalyzed conditions using α-phenylthioisobutyrophenone (Scheme 11), and nonbenzo-fused heteroarenes are thiolated using 2-methylthiothiazole (Scheme 45) [78]. The former thiolating reagent is more reactive than disulfides and α-thioketones because the less acidic nature of the 2-proton of 2-(methylthio)thiazole causes the chemical equilibrium to favor the formation of thiolated nonbenzo-fused heteroarenes. The reaction of 3-phenylthiazole and 2-(methylthio) benzothiazole (3 equivalents) in the presence of RhH(PPh_3_)_4_ (10 mol%) and 1,3-(dicyclohexylphosphino)-1,3-propane (dcypp) (20 mol%) in refluxing 1,2-dichlorobenzene for 3 h gave 2-methylthio-3-phenylthiazole in 74% yield. This reaction is under chemical equilibrium, and removal of volatile thiazole increased the yield. The reaction is applicable to various substituted thiazoles and oxazoles. 

Rhodium-catalyzed organothiolation reactions of heteroarenes and related compounds using disulfides [79,80,81,82,83,84,85,86,87,88,89] and sulfur [90] have recently been reported. These methods employ stoichiometric or substoichiometric amounts of copper(II) or silver(I) salts. The strong metal oxidizing reagents are considered to regenerate higher oxidation states of rhodium complexes and produce copper(I) salts or copper/silver metals as byproducts. The present reaction is characterized by simple transfer of organothio groups without using such metal reagents, which is achieved by judicious choice of organothio donor. A strong metal oxidizing reagent is an equivalent of a large amount of energy, and a related discussion will be provided in Section 8 on the use of the strong metal base of sodium hydroxide (Scheme 47). 

**Scheme 46:**
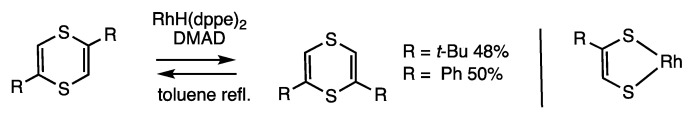
Exchange reaction of 1,4-dithiines.

2,5-Disubstituted 1,4-dithiines undergo rearrangement to provide 2,6-disubstituted derivatives, which involves the cleavage of two C-S bonds (Scheme 46) [91]. The reaction requires rhodium catalysis and is under chemical equilibrium. When 2,5-di(*t*-butyl)-1,4-dithiine was treated with RhH(dppe)_2_ (10 mol%) and dimethyl acetylenedicarboxylate (DMAD) (3 equivalents) in refluxing toluene for 24 h, 1,6-di(*t*-butyl)-1,4-dithiine was obtained in 48% yield, along with the starting material in 51% recovery yield. The role of acetylene was to stabilize the intermediate rhodium complex. Under forced conditions of 150 °C without a solvent, one of the olefin moieties in the starting material is exchanged with DMAD. The formation of a rhodacycle intermediate and the involvement of the thio-Diels–Alder reaction are proposed to occur.

Organosulfur compounds can be transformed into other organosulfur compounds and related organic compounds by the rhodium-catalyzed method owing to the reversible nature of the reactions. Such manipulation provides a diversity of derivatives starting from a single organosulfur compound by changing their cosubstrates.

## 8. Conclusions

### 8.1. Mechanisms of Rhodium-Catalyzed Substitution and Insertion Reactions of Disulfides 

**Figure 7:**
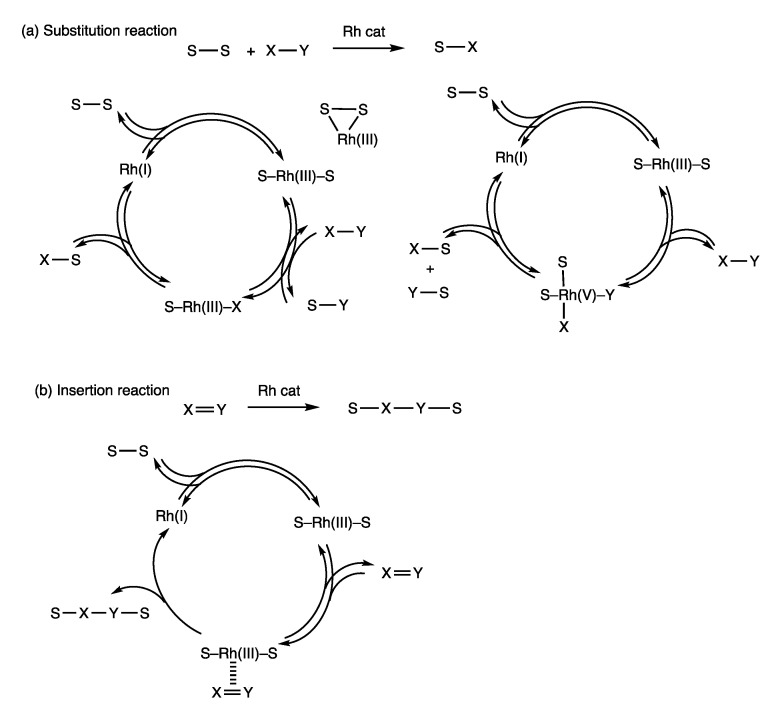
Mechanistic models of rhodium-catalyzed (**a**) substitution reactions and (**b**) insertion reactions with disulfides.

Rhodium complexes catalyze the cleavage of disulfide S-S bonds and transfer of organothio groups to other organic compounds, which provides various organosulfur compounds. Mechanistic models of substitution reactions and insertion reactions are shown below (Figure 7). A low-valent Rh(I) complex undergoes oxidative addition with a disulfide to provide a S-Rh(III)-S complex (Figure 7a). Such oxidative addition reactions of disulfides are known [92,93,94]. Formation of dithiorhodacycles by reactions of rhodium complexes and sulfur has also been reported [65,95]. Then, ligand exchange proceeds with a molecule possessing an X-Y bond to form an X-Rh(III)-S complex, and reductive elimination provides a product possessing a S-X bond with the regeneration of the Rh(I) complex. Alternatively, formation of a Rh(V) complex can be considered by subsequent oxidative addition of the S-Rh(III)-S complex with a molecule possessing an X-Y bond. 

Two reductive eliminations then provide two molecules containing X-S and Y-S bonds, which are accompanied by regeneration of the Rh(I) complex. Formation of Rh(V) complexes has been reported [96,97,98,99]. A mechanistic model of the insertion reaction can also involve oxidative addition of a Rh(I) complex and a disulfide, which is followed by the transfer of two organothio groups to an unsaturated molecule possessing an X=Y bond to form a product possessing a S-X-Y-S group with the regeneration of the Rh(I) complex (Figure 7b). 

Involvement of chemical equilibria is a notable feature of the present rhodium-catalyzed substitution reactions using disulfides. The above mechanistic model shows reversible nature in the oxidative addition of a rhodium(I) complex and a disulfide (Figure 7a). In addition, ligand exchange of a S-Rh(III)-S complex possessing an X-Y molecule is reversible; reductive elimination of an X-Rh(III)-S complex to provide a product with a S-X bond may be reversible. Such a sequence of reversible reactions involving organosulfur–rhodium complexes provides a chemical equilibrium. Disulfide exchange reactions described in Section 2 support the reversible mechanistic model.

Insertion reactions can also involve reversible oxidative addition of a Rh(I) complex and a disulfide (Figure 7b). Subsequent insertion of an X=Y molecule may be irreversible because reactions of unsaturated compounds to form saturated compounds are generally exothermic. 

It should be noted that the formation of Rh-S bonds can be reversible, which may be a basis for the rhodium-catalyzed synthesis of organosulfur compounds described in this article. This is in contrast to a general thought that chemical bonds between transition metals and sulfur atoms are very strong and make such catalysis difficult. 

### 8.2. Rhodium-Catalyzed Synthesis of Diverse Organosulfur Compounds

The rhodium-catalyzed synthetic method developed in this study provides organosulfur compounds with diverse structures; the development of highly active catalysts and design of exothermic reactions have been critical for this method. The synthesis employs disulfides and sulfur possessing S-S bonds, which are stable and readily available. Rhodium complexes cleave S-S bonds and promote substitution and insertion reactions with various organic compounds. 

**Scheme 47:**
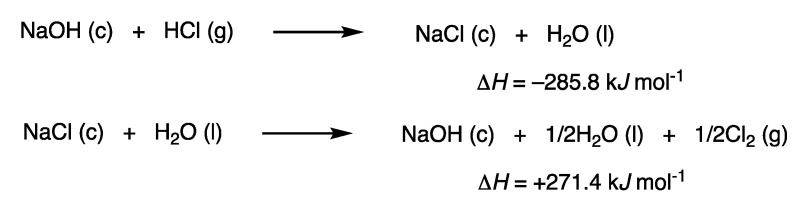
Thermodynamic analysis of the use of sodium hydroxide.

None of these reactions employ bases or organometallic reagent. This feature is in contrast to conventional syntheses of organosulfur compounds using thiolate anions and organohalogen compounds, which rely on the exothermic nature of reactions owing to the neutralization of hydrogen halides. Such a reaction involving rhodium catalysis was also reported [100]. In this context, the role of a base is considered, for example, in a reaction generating hydrogen chloride, which is a substitution reaction of an alkyl chloride and a thiol. Neutralization of hydrogen chloride with sodium hydroxide to form sodium chloride is strongly exothermic, ΔH = −285.8 kJ mol^−1^, as calculated from the heat of formation (Scheme 47). The regeneration of sodium hydroxide from sodium chloride by electrolysis requires a large amount of energy: ΔH = +271.4 kJ mol^−1^. No base is incorporated in the product, and the neutralization–regeneration cycle consumes energy to make the reaction exothermic. Metal halides are not easy to recover and reuse, and to do so requires a large amount of energy. A strong base is an equivalent of a large amount of energy; for example, the production of sodium hydroxide from sodium chloride (1 × 10^10^ kWh in 2017) [101] consumes 1% of all electricity in Japan (8 × 10^11^ kWh in 2015) [102]. The atom economy is often employed to analyze the efficiency of a synthetic chemical reaction [103]. The formation of metal halides can provide a favorable atom economy because their molecular weights are small. However, it is also critical to consider energy, and the reuse of metal halides requires a vast amount of energy.

**Figure 8:**
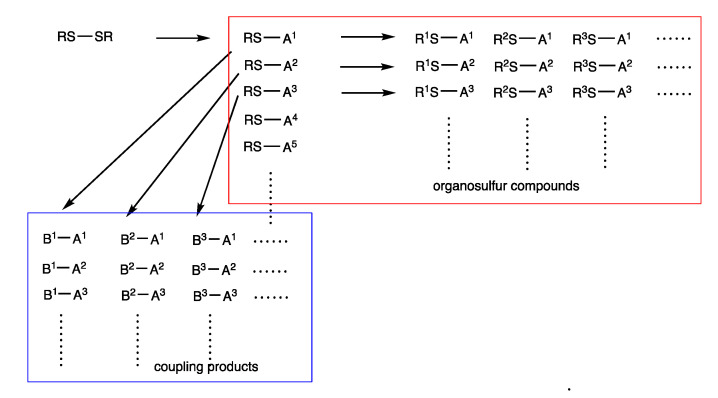
Synthesis of diverse organosulfur compounds and coupling products by rhodium-catalyzed reactions of disulfides.

Another notable aspect of the syntheses presented herein is the provision of diverse organic compounds that are structurally related. This feature is derived from the use of disulfides (RS-SR) and sequential substitution reactions (Figure 8). When a rhodium-catalyzed reaction converts one SR group with **A**, a series of products RS-**A**^1^, RS-**A**^2^, RS-**A**^3^, RS-**A**^4^, … are produced. Then, the rhodium-catalyzed organothio exchange reaction of RS-**A**^1^ provides diverse organosulfur compounds R^1^S-**A**^1^, R^2^S-**A**^1^, R^3^S-**A**^1^, …, as described in Section 2, Section 3 and Section 6. Using the reversible nature of the rhodium-catalyzed reactions, we can substitute the RS group in RS-**A**^1^ with **B**^1^, which provides coupling products **B**^1^-**A**^1^, **B**^1^-**A**^2^, **B**^1^-**A**^3^, …. Rhodium catalysis then provides other coupling products **B**^2^-**A**^1^, **B**^2^-**A**^2^, **B**^2^-**A**^3^, …, as described in Section 7. Thus, organothio groups derived from disulfides RS-SR have dual roles. One is as a part of organosulfur compounds R^n^S-**A**^m^; the other is as a leaving group to provide coupling products **B**^n^-**A**^m^. This chemistry provides a systematic method to construct a library of compounds in an energy-saving manner.

### 8.3. Chemical Reaction Network System

**Figure 9:**
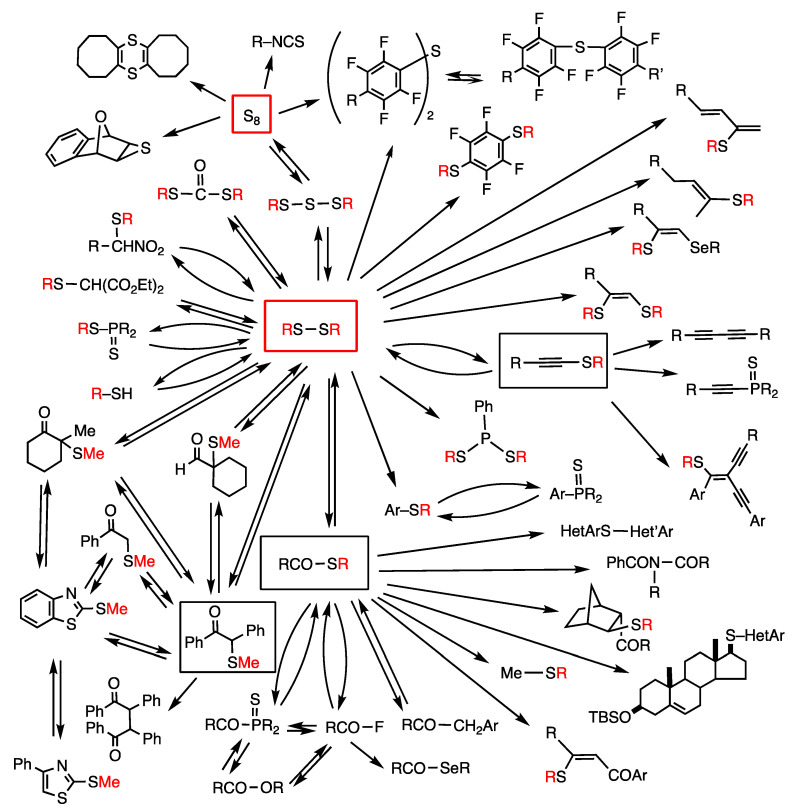
Schematic presentation of a rhodium-catalyzed chemical reaction network using disulfide and sulfur. Straight arrows pointing in opposite directions show chemical equilibria in closed systems, and curved arrows pointing in opposite directions show interconversion reactions in open systems. Red R letters are derived from organic groups in disulfides. Squares contain important synthetic intermediates.

The rhodium-catalyzed synthesis of organosulfur compounds involves reversible reactions, which are chemical equilibria and interconversion reactions (Figure 9). Chemical equilibrium in closed systems involves a comparable thermodynamic stability of substrates and products, along with a low energy barrier (Figure 4). Such reactions are shown by straight arrows pointing in opposite directions. Interconversion reactions in open systems involve the addition of appropriate organic cosubstrates and the formation of coproducts, which inverts the relative thermodynamic stability of substrates and products. The reactions can then proceed in either direction, as shown by curved arrows pointing in opposite directions. The summary of this work provides a chemical reaction network with many reversible reactions, in which substrates and products are mutually related (Figure 9). The product of a reaction can be a substrate in the next or distantly located chemical reactions. This chemical reaction network, however, is a simplified model based on the reactions of disulfide and sulfur, and the real network is multidimensional. As examples of such networks, we have previously proposed an acylation reaction network [49] and a C-H thiolation reaction network [29].

Biological systems employ complex chemical reaction networks with energy-saving characteristics. Chemical equilibria can be controlled by flow systems in biological cells, and interconversion reactions can be controlled by external energy using ATP and ADP to produce exothermic reactions. Biological reaction networks are further controlled by enzymatic catalysis [6,7]. The chemical reaction network herein can be a model of biological systems involving many chemical equilibria and interconversion reactions, although biological systems are much more well-organized and functional.
